# Recycling of Brewer’s Spent Grain as a Biosorbent by Nitro-Oxidation for Uranyl Ion Removal from Wastewater

**DOI:** 10.1021/acsomega.1c00589

**Published:** 2021-07-19

**Authors:** Yi Su, Marco Wenzel, Silvia Paasch, Markus Seifert, Wendelin Böhm, Thomas Doert, Jan J. Weigand

**Affiliations:** † Chair of Inorganic Molecular Chemistry, TU Dresden, 01062 Dresden, Germany; ‡ Chair of Bioanalytical Chemistry, TU Dresden, 01062 Dresden, Germany; § Chair of Food Chemistry, TU Dresden, 01062 Dresden, Germany; ∥ Chair of Inorganic Chemistry II, TU Dresden, 01062 Dresden, Germany

## Abstract

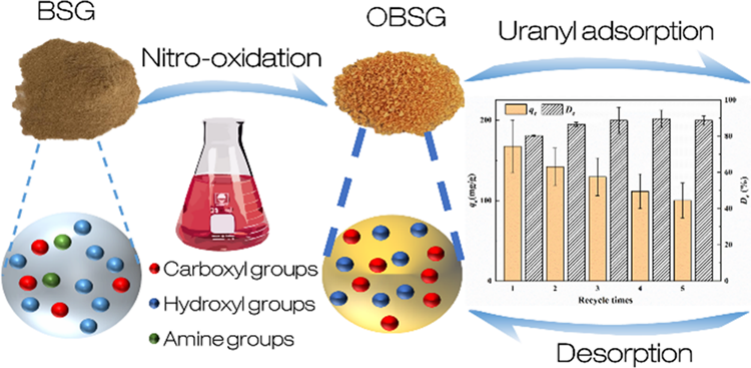

Developing biosorbents derived from agro-industrial biomass is considered as an economic and sustainable method for dealing with uranium-contaminated wastewater. The present study explores the feasibility of oxidizing a representative protein-rich biomass, brewer’s spent grain (BSG), to an effective and reusable uranyl ion adsorbent to reduce the cost and waste generation during water treatment. The unique composition of BSG favors the oxidation process and yields in a high carboxyl group content (1.3 mmol/g) of the biosorbent. This makes BSG a cheap, sustainable, and suitable raw material independent from pre-treatment. The oxidized brewer’s spent grain (OBSG) presents a high adsorption capacity of U(VI) of 297.3 mg/g (*c*_0_(U) = 900 mg/L, pH = 4.7) and fast adsorption kinetics (1 h) compared with other biosorbents reported in the literature. Infrared spectra (Fourier transform infrared), ^13^C solid-state nuclear magnetic resonance spectra, scanning electron microscopy, energy-dispersive X-ray spectroscopy, and thermogravimetric analysis were employed to characterize the biosorbents and reveal the adsorption mechanisms. The desorption and reusability of OBSG were tested for five cycles, resulting in a remaining adsorption of U(VI) of 100.3 mg/g and a desorption ratio of 89%. This study offers a viable and sustainable approach to convert agro-industrial waste into effective and reusable biosorbents for uranium removal from wastewater.

## 1 Introduction

Uranium is one of the most widely applied radioactive elements commonly used as a source for nuclear fuel.^1^ It is also a potential important catalyst with practical advantages not found among transition metals.^2^ However, uranium presents high radiologic and chemical toxicity^3^ and can be mobile in the water system and migrate through food chains,^4^ imposing severe threats to the environment and the human health. In particular, uranium presents renal, developmental, and reproductive toxicity and causes diminished bone growth and DNA damage, as obtained from experimental animal studies and human epidemiology.^5^ Numerous adsorbents have been explored to remove uranium from the water environment, such as mesoporous carbon,^6^ graphene oxides,^7^ hydrochar,^8^ chitosan,^9^ amidoxime-based materials,^10^ and functional fibrous material-based adsorbents.^11^ Natural organic raw materials are the most attractive materials for green adsorbent production, and the diversity of raw materials of biosorbents has been increased rapidly, including hazelnut shells,^12^ chitosan,^13^ nanocellulose,^14^ starch,^15^ saw dust,^16^ etc. These biosorbents possess several advantages such as very low cost, easy to functionalize, and the possibility of further volume decrease by pyrolysis.^17^ Furthermore, the biocompatibility and non-toxicity of biosorbents are safe for animal and human health when applying in the water environment.^18,19^

Oxidation of cellulosic materials is of great interest in developing effective adsorbents from biomass due to the obtained high content of carboxyl groups that have a high affinity toward metal ions.^20^ According to the literature,^21^ nitrogen oxides, permanganates, peroxides, and stable and non-persistent nitroxyl radicals (TEMPO and PINO) have been applied for the oxidation of cellulose. For example, Ma et al.^22^ have obtained ultrafine cellulose nanofibers from wood pulp by TEMPO oxidation with a carboxyl group content of 1.4 mmol/g and a U(VI) adsorption capacity of 167 mg/g. Nevertheless, the widely applied nitroxyl radicals present some obvious drawbacks like high cost and toxic regents and are only effective on cellulose components with little impurities.^21^ An early work by Kumar and Yang^23^ reports the combination of H_3_PO_4_/HNO_3_-NaNO_2_ as oxidants to produce carboxycellulose from untreated biomass, which is termed as “nitro-oxidation”, providing a simple and selective oxidation way without transition metals as catalysts. Carboxycellulose nanofiber obtained from jute fiber employing this method shows a high affinity toward U(VI) with clear precipitation (pH = 7, *c*_0_(U) = 2120 mg/L). The reported adsorption capacity is higher than expected considering the available carboxyl groups of the nanofibers. This is mainly due to the aggregation of the nanofibers and the mineralization of uranyl ions forming uranyl hydroxide crystals rather than a result of a simple adsorption process.^24^ This makes it an extreme and rare example employing nitro-oxidation cellulose nanofibers for uranyl ion adsorption. Contradicting to the fact that most of the studies regarding cellulose oxidation are only focused on pure cellulose or commercial fibers, an extensive amount of cheap and easily available biomass from agricultural or industrial waste streams is still waiting for exploration. Furthermore, only a few studies have discussed desorption and reusability of oxidized cellulose materials. This raises the problem that non-renewable biosorbents would increase the operation cost and waste production of the adsorption process, thus undermining the economic and ecologic benefits of the biosorbents.^25^

Brewer’s spent grain (BSG) is the main byproduct from the beer brewery industry with a global production of 39 million tons per year. BSG is produced all year round in all kinds of breweries, which makes it a cheap, widely available, and continuously accessible raw material for biosorbents.^26^ Despite the large produced amount, BSG has received little attention as a valuable commodity, and its disposal is often problematic to the environment.^27^ Because of its low cost and abundant surface functional groups, several studies have been devoted to modify BSG to improve its adsorption performance, including heat conversion and chemical modification. The production of active carbon^28^ and activated hydrochar^29^ are the most common heat conversion methods, however, with high energy consumption. In our previous study,^30^ hydrothermal treatment of BSG at low temperature (150 °C) without activation yields a biosorbent with an adsorption capacity of U(VI) of 220.6 mg/g. In addition, complicated chemical modifications such as esterification^31^ and thiol functionalization^32^ of BSG have also been reported. Considering the enormous amount of BSG generated continuously, it is still attractive to investigate alternative approaches with less energy consumption, less toxic chemical demands, and simpler processes to recycle BSG as an effective biosorbent and improve its potential for application.

It is reported from the literature^33^ that the occurrence and maintenance of a long-time stable foam generated by liberated nitrogen oxides in highly viscous H_3_PO_4_ are crucial for successful nitro-oxidation. Thus, it is speculated that BSG could be an ideal raw material for the nitro-oxidation of cellulose, which is rich in proteins and could produce and maintain foam during the oxidation process. To the best of our knowledge, there is no attempt to oxidize BSG and use it as a biosorbent in the literature. The goal of the present study is to demonstrate that BSG, as a representative biomass rich in protein and lignocellulose, is a low-cost and easily available source for effective U(VI) biosorbents. In the present study, aside from nitro-oxidation,^23^ H_2_O_2_ and KMnO_4_ have also been tested as oxidants, and only nitro-oxidation is proven to oxidize BSG successfully. Therefore, the nitro-oxidation method has been investigated thoroughly. The effects of the particle size of BSG on the nitro-oxidation, the chemical structure, functional groups, and thermal stability of oxidized BSG (OBSG) were studied and characterized. Adsorption properties, adsorption mechanisms, and the reusability of OBSG were also investigated to provide a complete picture of the potential of OBSG as biosorbent for U(VI) removal.

## 2 Results and Discussion

### 2.1 Oxidation of Brewer’s Spent Grain

**Figure 1:**
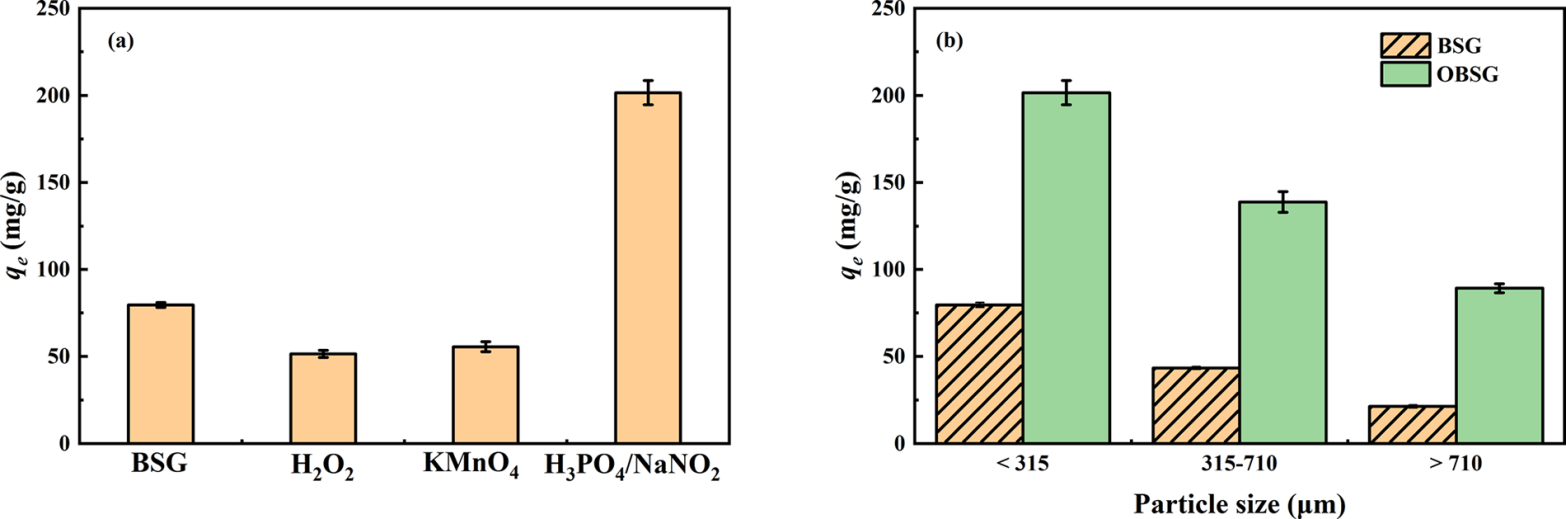
(a) Adsorption capacity of the U(VI) onto oxidized products using different oxidation methods (BSG < 315 μm) and (b) effect of the particle size on the adsorption capacity before and after nitro-oxidation. For adsorption, 2 mg of the adsorbent/2 mL of solution, *c*_0_(U) = 300 mg/L, pH = 4.7, 2 h, room temperature.

Aside from nitro-oxidation, H_2_O_2_^34,35^ and KMnO_4_^36^ were also tested as oxidizing agents in the present study to explore an appropriate oxidation method for BSG. The U(VI) adsorption capacity employing the obtained products (*c*_0_(U) = 300 mg/L) is shown in Figure 1a, and the FT-IR spectra of the products are provided in Figure S1. Only the product obtained from the nitro-oxidation method (H_3_PO_4_/NaNO_2_ as oxidants) shows an increased adsorption capacity for U(VI). The rise in the adsorption capacity from 79.6 mg/g for BSG to 201.6 mg/g for OBSG shows the successful conversion of BSG and a significant enhancement of the U(VI) adsorption. The other studied oxidants result in materials with a lower adsorption capacity than BSG, which may be due to the loss of surface functional groups during the non-selective oxidation. This is also confirmed by the analysis of the Fourier transform infrared (FT-IR) spectra (Figure S1). Only OBSG derived from nitro-oxidation shows an increased intensity of the absorption band attributed to the C=O stretching vibration of the −COOH groups (1732 cm^–1^), while applying other oxidation methods results in a decreased intensity of this absorption band. Therefore, nitro-oxidation is proven as an effective oxidation method for BSG. Mixing of H_3_PO_4_ and NaNO_2_ results in the release of nitroxonium ions (NO^+^) in the presence of excess acid, which is able to selectively oxidize the primary hydroxyl group (−CH_2_OH) of cellulose at the C6 position to carboxyl groups.^23,24^ The presence of H_3_PO_4_ may also act as a good swelling agent for cellulose and aid to remove impurities (lignin, protein, and hemicellulose) from untreated BSG, which favors the selective oxidation of cellulose.

The results in Figure 1b show the adsorption capacity (*c*_0_(U) = 300 mg/L) of BSG with different particle sizes and OBSG obtained from different particle sizes of BSG. The adsorption capacity of BSG increases as the particle size becomes smaller, from 21.3 mg/g (>710 μm) to 79.6 mg/g (<315 μm). This is attributed to the fact that the smaller particle fraction of BSG contains more protein and starch-rich components with abundant functional groups such as hydroxyl groups and carboxyl groups,^37^ resulting in the increased adsorption capacity. After oxidation, the adsorption capacity of OBSG obtained from different particle sizes of BSG also increases as the particle sizes decrease from >710 μm (89.1 mg/g) to 315–710 μm (138.8 mg/g) and <315 μm (201.6 mg/g). This rise in the adsorption capacity of OBSG may be attributed to the increase in the specific surface area with the decreasing particle size. It allows BSG to swell more easily and the availability of active sites for effective oxidation increases.^38^ As the OBSG obtained from the smallest fraction (<315 μm) shows the highest adsorption capacity of 201.6 mg/g, this fraction (<315 μm) of BSG and OBSG was used for the detailed adsorption studies and further characterizations.

### 2.2 Characterization

#### 2.2.1 FT-IR and Solid-State NMR Spectra

**Figure 2:**
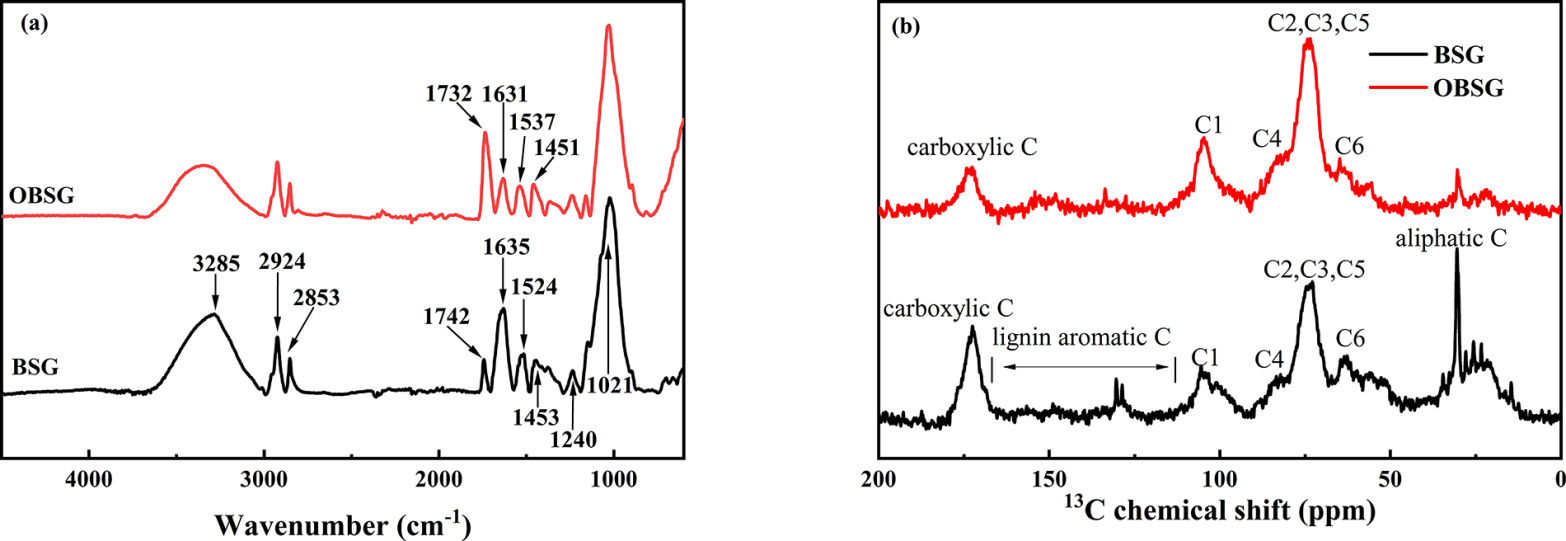
(a) FT-IR spectra and (b) ^13^C CP/MAS solid-state NMR spectra of BSG and OBSG.

FT-IR spectra provide important information about the structure and major functional groups of BSG and OBSG, as shown in Figure 2a. Briefly, for both BSG and OBSG, the broad absorption bands at 3200–3400 cm^–1^ could be assigned as the overlapping of O–H and N–H stretching vibrations that are abundant in lignocellulose and proteins. The absorption bands around 2900 and 2800 cm^–1^ are assigned to the antisymmetric and symmetric stretching vibrations of −CH_2_ groups from cellulose and hemicellulose, and the absorption bands at 1240 and 1021 cm^–1^ are attributed to the C–N and C–O–C stretching vibrations, respectively.^39^ These show that an abundant number of hydroxyl groups remain on the surface of OBSG, and the carbon chain of cellulose is unaffected upon oxidation. As for BSG, the absorption band at 1742 cm^–1^ is attributed to the C=O vibration of −COOH groups. In the spectrum of OBSG, this band shifts to 1732 cm^–1^ with a significant increase in intensity, showing the rise in the number of carboxyl groups due to oxidation.^35^ The attribution of this band to the C=O vibration of −COOH groups is confirmed for both adsorbents by shifting the absorption bands down by 3 cm^–1^ upon D^+^ labeling (Table S1 and Figure S2). The absorption band at 1635 cm^–1^ in the BSG spectrum is associated with the overlapping of the −COO^–^ antisymmetric stretching vibration and the protein-related bonds (amide I groups), while the absorption band at 1524 cm^–1^ is assigned to the amide II groups in proteins. The intensity of the absorption band at 1635 cm^–1^ decreases for OBSG, indicating the loss of protein during the oxidation process. In addition, the symmetric stretching vibration of −COO^–^ groups could still be observed at 1451 cm^–1^ (1453 cm^–1^ for BSG).^40^

In addition to FT-IR spectra, the ^13^C CP/MAS solid-state NMR spectra of BSG and OBSG also present structural information about the biosorbents. As shown in Figure 2b, OBSG presents a structure similar to the oxidized cellulose reported in the literature.^41^ For example, the resonances at 105 ppm (C1), 84 ppm (C4), 73 ppm (C2, C3, and C5), and 64 ppm (C6) are associated with the carbon backbone of cellulose and the resonance at 173 ppm is attributed to carboxyl groups.^42^ In the BSG spectrum, the resonances at 120–160 and 30 ppm, which are assigned to the lignin aromatic C and aliphatic C from sugar chains,^43^ are more pronounced than those in the OBSG spectrum. This is probably due to the removal of impurities during oxidation.

#### 2.2.2 Chemical Composition and Functional Groups

**Figure 3:**
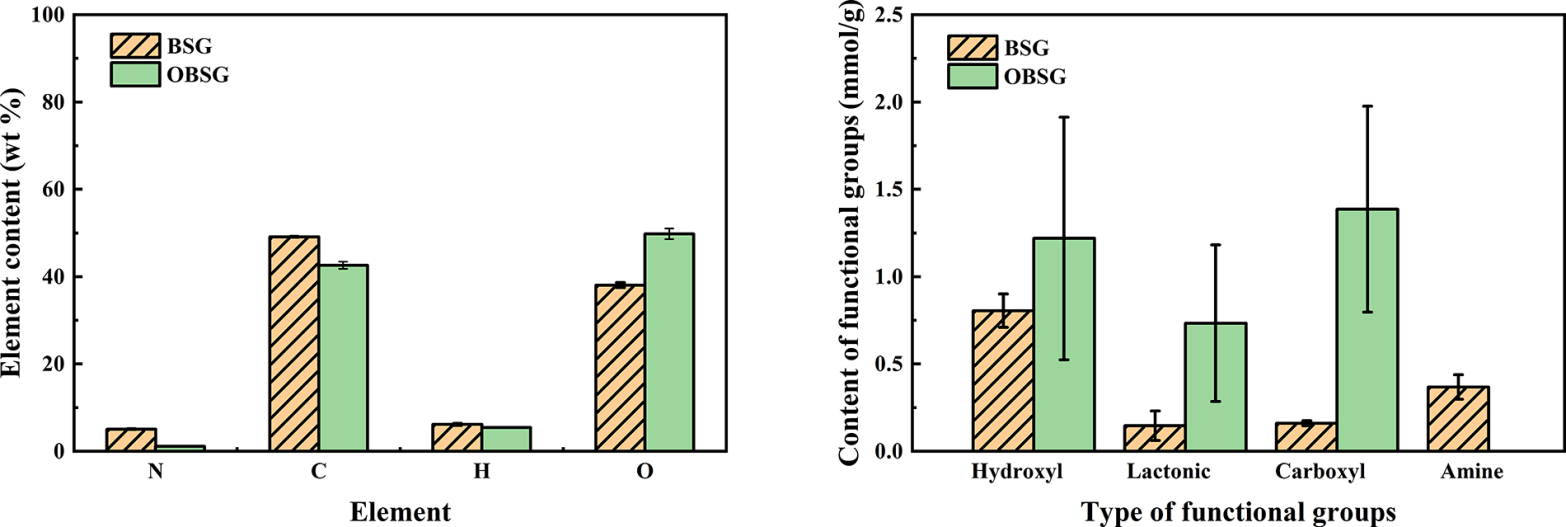
(a) Elemental analysis and (b) content of functional groups of BSG and OBSG.

Elemental analysis of BSG and OBSG shows changes in the C, H, N, and O contents in weight percentage (Figure 3a and Table S2). After oxidation, the content of N decreases from 5.1 to 1.1 wt %; thus, the protein content is estimated to decrease from 29.5 to 6.4 wt %. This is consistent with the obtained decrease in the absorption band intensity of the amide I groups at 1635 cm^–1^ in the FT-IR spectra and shows that most of the protein is removed during the oxidation. However, the presence of protein during oxidation is important. It could act as a surfactant and form a large quantity of foam when N_2_O_3_ is released from the reaction of H_3_PO_4_ and NaNO_2_. According to the literature,^33^ the high specific surface area of the foam and excessive pressure inside are crucial for a successful oxidation. The content of C decreases from 49.1 wt % (BSG) to 42.6 wt % (OBSG), and the content of H decreases from 6.1 wt % (BSG) to 5.4 wt % (OBSG). In addition, the O content calculated by the difference increases from 38.0 wt % (BSG) to 49.8 wt % (OBSG). Furthermore, the phosphorous content of OBSG (760 ± 30 mg/kg) is significantly lower than that of BSG (5284 ± 3 mg/kg) (see Table S3) despite the usage of phosphoric acid during the oxidation. This indicates that the increased adsorption capacity of OBSG is not due to the presence of residual phosphoric acid in OBSG samples, which would have favored the precipitation of uranyl phosphate. Instead, the increased content of carboxyl groups mainly contributes to the enhanced adsorption capacity.

The determined content of the functional groups involved in adsorption is shown in Figure 3b. Obviously, the quantity of carboxyl groups increases significantly from 0.15 to 1.3 mmol/g, confirming the successful oxidation of BSG. This is comparable to the literature^22,44^ that the carboxyl group content of oxidized cellulose ranges from 0.4 to 1.4 mmol/g. Meanwhile, the content of free amine groups drops from 0.4 mmol/g to 0, which is also consistent with the removal of protein under the applied oxidation conditions. In addition, the contents of hydroxyl groups (0.8 mmol/g BSG and 1.2 mmol/g OBSG) and lactonic groups (0.2 mmol/g BSG and 0.7 mmol/g OBSG) increase upon oxidation despite the conversion of primary hydroxyl groups to carboxyl groups. Presumably, the overall change of the material composition, such as the removal of protein, leads to the increase in oxygen-containing functional groups. In particular, the increased number of carboxyl groups would be of great advantage for uranium adsorption.

#### 2.2.3 Thermogravimetric Analysis

**Figure 4:**
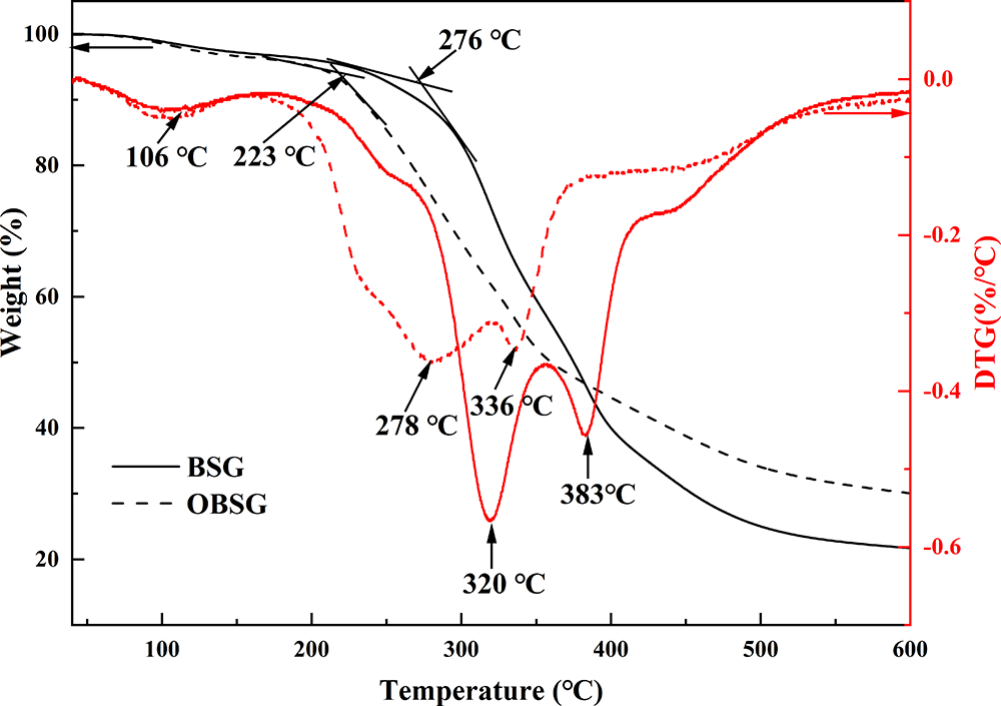
TG and DTG of BSG and OBSG (20 °C/min, He atmosphere).

Thermogravimetric (TG) analysis is employed to explore the thermal degradation properties of the adsorbents. The thermogravimetric–derivative thermogravimetric (TG-DTG) curves in Figure 4 show the four-step decomposition of both BSG and OBSG. Both samples display mass losses of 3.0 wt % (BSG) and 3.6 wt % (OBSG) between 40 and 162 °C with a maximum decomposition temperature (DTG peak) at 106 °C, which mainly comes from the evaporation of water. As for BSG, the onset temperature is 276 °C, and it undergoes a second step with a DTG peak at 320 °C. During the second step ranging from 162 to 358 °C, 40.4 wt % of the BSG mass is lost due to the decomposition of protein and degradation of hemicellulose (from 220 °C) and cellulose (from 310 °C).^45^ In addition, lignin is also gradually decomposed over a wide temperature range from 180 to 550 °C.^46^ Further decomposition of hemicellulose, cellulose, and lignin results in the third step with a DTG peak at 383 °C. When the temperature exceeds 439 °C, the fourth step occurs with the decomposition of lignin and the carbonation process,^47^ resulting in a residue of 21.7 wt %. In contrast, a somewhat lower onset temperature of 223 °C is determined for OBSG, which is probably due to the increase in anhydroglucuronic acid units on its surface with lower thermal stability.^48^ This also results in the shift of the second step to a lower temperature range (162–321 °C) with a DTG peak at 278 °C, which is derived from the earlier degradation of oxidized cellulose. In addition, the acid hydrolysis and mass loss of lignin and hemicellulose also shift the third step to a slightly lower temperature with a DTG peak at 336 °C.^49^ Although OBSG demonstrates less thermal stability than BSG at the early heating stage, it has a higher residue of 30.1 wt % after decomposition. Presumably, the formation of a carbonaceous layer on the surface of OBSG delays the thermal decomposition.^50^

### 2.3 Adsorption Studies

#### 2.3.1 Effect of Equilibrium pH

**Figure 5:**
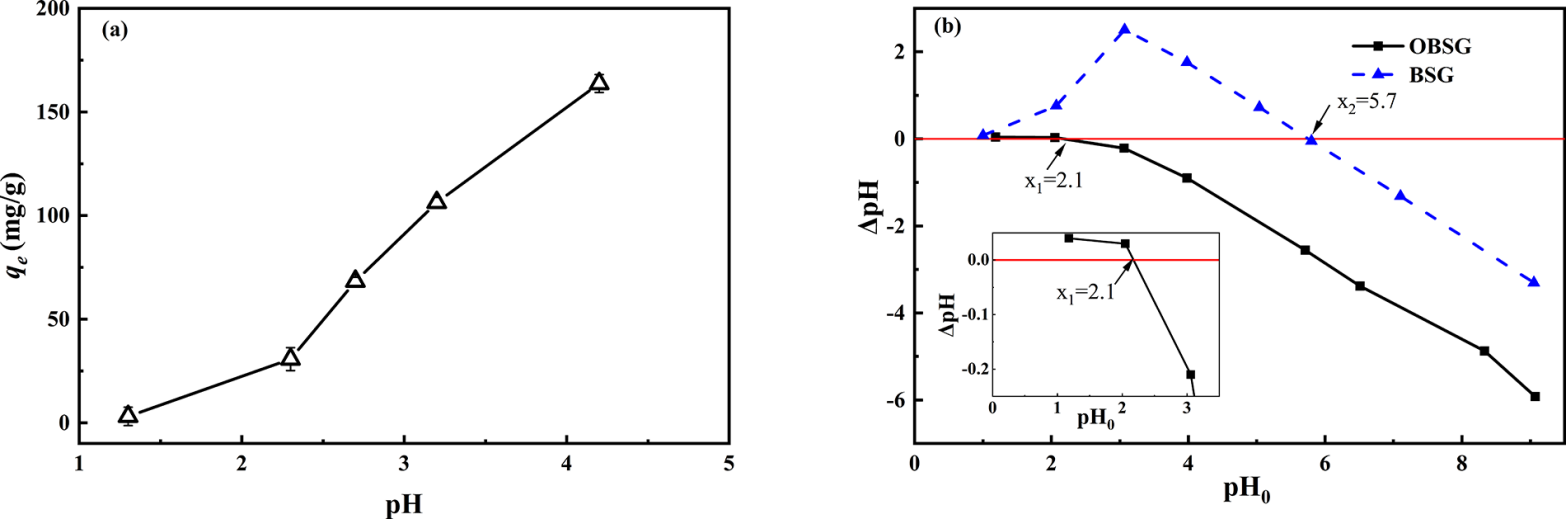
(a) Effect of equilibrium pH on the adsorption capacity. For adsorption, 2 mg of the adsorbent/2 mL of solution, *c*_0_(U) = 300 mg/L, 1 h, room temperature; (b) determination of pH_pzc_ of BSG and OBSG.

The pH of metal solution is a crucial factor for adsorption because it determines the speciation of metal ions and the surface properties of the adsorbents. The effect of equilibrium pH on the U(VI) adsorption capacity of OBSG is depicted in Figure 5a, and the determination of the point of zero charge (pH_PZC_) is shown in Figure 5b. A distribution diagram of uranyl acetate solution that contains 300 mg/L (1.26 mM) U(VI) as a function of pH has been calculated using Visual MINTEQ 3.1 software^51^ and is shown in Figure S3. The saturation index of UO_2_(OH)_2_ is >0 when the pH increases over 5, indicating that the solution is supersaturated and precipitation of UO_2_(OH)_2_ would occur when pH > 5. Therefore, the pH dependency experiments were carried out in the pH range of 1–5 to prevent precipitation. As there is no pH buffer in the current adsorption system, the equilibrium pH is not equal to the initial pH of the uranium solution. This is because of the H^+^ release from the carboxyl groups of OBSG upon the coordination of U(VI).^52^ To provide comparable information with other studies^7,9^ that discuss the initial pH of the uranium solution, both initial and equilibrium pH values are given in Table S4. As seen in Figure 5a, the adsorption capacity of OBSG increases as pH increases from 1 (3 mg/g) to 5 (163 mg/g). When the equilibrium pH < pH_PZC_ of OBSG (pH < 2.1), the electrostatic repulsion between the positively charged adsorbent surfaces and UO_2_^2+^ species hinders the adsorption process.^53^ As the equilibrium pH rises over the pH_PZC_, the surface charge of OBSG becomes negative due to the deprotonation of carboxyl groups, causing strong electrostatic attraction toward UO_2_^2+^. In addition, the strongly negative-charged surface of OBSG prevents the aggregation of OBSG particles, which offers more opportunities for the interaction between UO_2_^2+^ and the surface functional groups. At the same time, the species of uranyl ions in solution have changed. As shown in Figure S3, UO_2_^2+^ is predominant at pH < 2.5, and with increasing the pH from 2.5 to 5, hydrolyzed species such as (UO_2_)_2_(OH)_2_^2+^, UO_2_OH^+^, and (UO_2_)_3_(OH)_5_^+^ occur in coexistence with UO_2_^2+^.^54^ Generally, despite the same charge, the solid surface has a higher affinity toward the hydrolyzed species (UO_2_)_2_(OH)_2_^2+^ than UO_2_^2+^.^53,55^ Furthermore, the monocationic species UO_2_OH^+^ and (UO_2_)_3_(OH)_5_^+^ have a reduced electrostatic effect due to their low charge, but the ion-exchange ratio between the carboxyl groups and the uranyl species decreases from 2:1 to 1:1. Thus, more functional groups are available for interactions toward uranyl ions in the adsorption process. As a result, a considerable adsorption capacity (>30 mg/g) of U(VI) for an equilibrium pH higher than 2 is observed.

#### 2.3.2 Adsorption Kinetics

**Figure 6:**
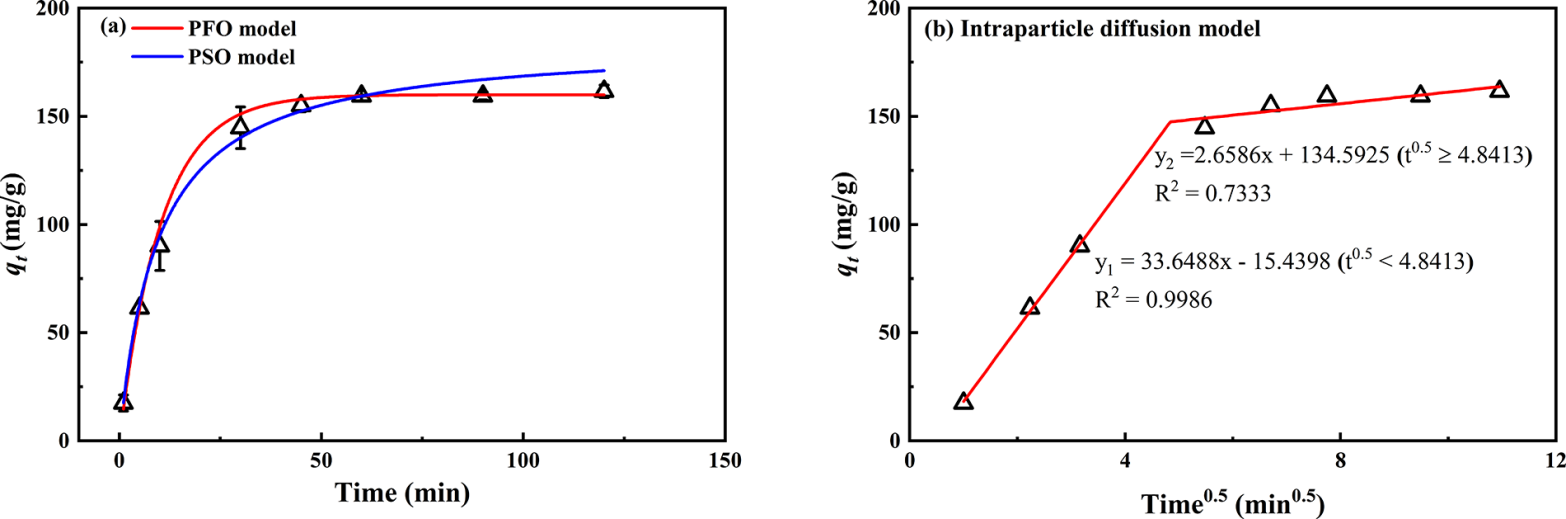
(a) Adsorption kinetics of U(VI) onto OBSG and non-linear fitting of pseudo-first-order (PFO) and pseudo-second-order (PSO) kinetic models. For adsorption, 2 mg of OBSG/2 mL of solution, *c*_0_(U) = 300 mg/L, pH = 4.7, rotation speed = 60 rpm, room temperature; (b) piecewise linear regression of the intraparticle diffusion model.

The effect of contact time on the adsorption of U(VI) onto OBSG is illustrated in Figure 6a. At the early stage (0–30 min), the amount of U(VI) adsorbed on OBSG increases rapidly due to the large concentration gradient between liquid and solid phases and the large number of vacant adsorption sites on the adsorbent surface.^56^ After 30 min, the adsorption rate slows down as the concentration gradient decreases, the adsorption sites are occupied, and the adsorption reaches equilibrium at 60 min with an adsorption amount of 159.5 mg/g. It is generally accepted that the adsorption dynamics consists of three steps, namely, external diffusion, internal diffusion, and the adsorption of adsorbates onto the active sites of the adsorbents.^57^ Herein, non-linear fittings of pseudo-first-order (eq 1) and pseudo-second-order (eq 2) kinetic models have been used to describe the diffusion step and the adsorption onto active sites, respectively^58^
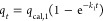
1
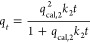
2where *q_t_* (mg/g) is the adsorption capacity at time *t* (min) and *q*_cal,1_ (mg/g) and *q*_cal,2_ (mg/g) are the equilibrium adsorption capacities estimated by the pseudo-first-order and pseudo-second-order kinetic model, respectively; *k*_1_ (min^–1^) and *k*_2_ (g·mg^–1^·min^–1^) are the rate constants of pseudo-first-order and pseudo-second-order kinetic models, respectively. Several statistical parameters (*R*^2^, residual sum of squares (RSS), and χ^2^)^58^ and well-known statistical methods (F test, Akaike’s information criterion (AIC), and Bayesian information criteria (BIC) test)^59^ were applied to evaluate the performance of the kinetic models. The fitting results and kinetic parameters are summarized in Table 1, and the detailed statistical evaluations are given in Table S5. The analyses show that the pseudo-first-order kinetic model presents better results than the pseudo-second-order kinetic model in terms of statistical parameters (smaller *R*^2^, RSS, and χ^2^) and the estimation of equilibrium adsorption capacity (a *q*_e,cal1_ of 160.0 mg/g compared with a *q*_e,cal2_ of 184.6 mg/g). In addition, all three statistical methods (AIC, BIC, and F test) give preferred results for the pseudo-first-order kinetic model (Table S5). Therefore, it can be concluded that the pseudo-first-order kinetic model could better fit the kinetic data, which indicates that the adsorption dynamics is controlled by the diffusion step.^60^ In addition, the intraparticle diffusion model (eq 3) has also been applied using piecewise linear regression as shown in Figure 6b to figure out exactly which diffusion step controls the adsorption process^59^
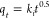
3where *k_i_* (mg/g min^0.5^) is the intraparticle diffusion parameter. According to the model, if the adsorption process is controlled by intraparticle diffusion, then the plot of *q_t_* versus *t*^0.5^ would be linear and pass through the origin.^59^ It is clear from Figure 6b that the plot is not linear and does not pass through the origin but could be divided into two linear regions with a breakpoint *t*^0.5^ = 4.8 min^0.5^. A possible explanation for this is that film diffusion controls the adsorption rate in the early stage; then, the intraparticle diffusion gradually takes control over the adsorption process.^61^ However, as OBSG is a non-porous material, the effect of intraparticle diffusion on the adsorption rate is expected to be small, indicated by a smaller *k*_*i*2_ value (2.6586) compared to the first linear region (*k*_*i*1_ = 33.6488) and the slow increase in adsorption after the breakpoint. As film diffusion is proposed to be the dominant rate-controlling step of the adsorption process, an additional kinetics study is performed trying to decrease this effect and increase the adsorption rate by increasing the rotation speed. As shown in Figure S4a, the adsorption of U(VI) on OBSG increases slightly at the first 10 min when increasing the rotation speed from 60 to 80 rpm. Furthermore, the kinetics data at 80 rpm are fitted with the kinetic models, as shown in Figure S4b, and the fitting results and statistical parameters are given in Tables S6 and S7. The analyses suggest that the pseudo-second-order kinetic model gives a better representation for the data at 80 rpm. This could indicate that the rate-controlling step of adsorption probably changes from film diffusion to the adsorption on active sites as the rotation speed is increased.^60^ However, for both rotation speeds, the adsorption process requires 60 min to reach the adsorption equilibrium, and the obtained difference in the adsorption capacity (159.5 mg/g for 60 rpm and 167 mg/g for 80 rpm) is within 5%. As the overall adsorption kinetics show no obvious improvement upon increasing the rotation speed, 60 rpm was chosen for the following experiments.

**Table 1:** Parameters of Kinetic Models^a^

pseudo-first-order kinetic model	*k*_1_ (min^–1^)	*q*_e,exp_ (mg/g)	*q*_e,cal1_ (mg/g)	*R*^2^	RSS	χ^2^
	0.0962	159.5	160.0	0.9994	3.07	0.51

pseudo-second-order kinetic model	*k*_2_ (g·mg^–1^·min^–1^)	*q*_e,exp_ (mg/g)	*q*_e,cal2_ (mg/g)	*R*^2^	RSS	χ^2^
	0.0006	159.5	184.6	0.9941	29.92	4.99

a For adsorption, 2 mg of OBSG/2 mL of solution, *c*_0_(U) = 300 mg/L, pH = 4.7, rotation speed = 60 rpm, room temperature.

#### 2.3.3 Adsorption Isotherm

**Figure 7:**
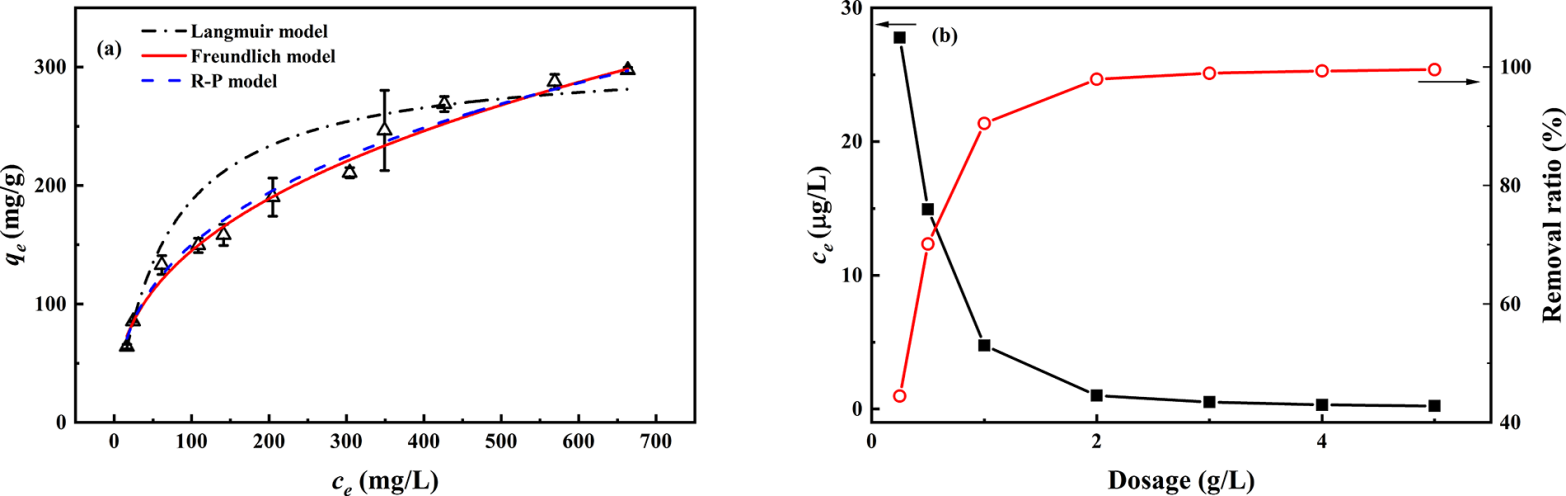
(a) Adsorption isotherm of U(VI) onto OBSG and nonlinear fitting curves of the Langmuir model, Freundlich model, and R-P model. For adsorption, 2 mg of OBSG/2 mL of solution, *t* = 1 h, pH = 4.6–4.7, room temperature; (b) calculation of the equilibrium concentration (*c*_e_, μg/L) and removal ratio (%) using a given initial U(VI) concentration of 50 μg/L according to the Freundlich model determined in panel (a).

The effect of initial metal concentration on the adsorption capacity of OBSG is shown in Figure 7, and the data are fitted by the Langmuir model (eq 4), Freundlich model (eq 5), and Redlich–Peterson (R-P) model (eq 6) taking into account the standard deviations^62^
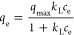
4where *c*_e_ (mg/L) is the equilibrium concentration of metal ions, *q*_e_ (mg/g) is the adsorption capacity, *q*_max_ (mg/g) is the maximum adsorption capacity estimated by the Langmuir model, and *k*_L_ (L/mg) is the Langmuir isotherm constant
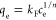
5where *k*_F_ ((mg/g) (L/mg)^1/*n*^) is the Freundlich isotherm constant related to adsorption capacity and *n* is the Freundlich isotherm constant related to adsorption intensity
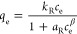
6where *k*_R_ (L/g) and *a*_R_ (L^β^/mg^β^) are the Redlich–Peterson isotherm constants and β is the Redlich–Peterson isotherm exponent. The fitting results and isotherm parameters are summarized in Table 2, and the detailed statistical evaluations are given in Table S8. The determined values show that the Langmuir model gives the worst fitting results with the lowest *R*^2^ (0.9739) and the highest error functions (RSS = 249 and χ^2^ = 27.7). In contrast, both the Freundlich model and the R-P model show good fitting results (*R*^2^ > 0.99) with very close values of the statistical parameters (Table 2) and overlapping simulated curves in Figure 7. According to the literature, if *a*_R_*c*_e_^β^ ≫ 1 (*a*_R_*c*_e_^β^ = 8–54 in the current case), then the R-P model can be approximated as the Freundlich model.^63^ To avoid over-parameterization, the F test, AIC, and BIC test were performed to compare the complex (R-P model) and simple models (Freundlich model). The results given in Table S8 illustrate that the R-P model is not essentially better than the Freundlich model; thus, the simpler Freundlich model should be used to describe the adsorption isotherm. This indicates that the adsorption of U(VI) onto OBSG is a multilayer adsorption on heterogeneous surfaces.^64^

**Table 2:** Parameters and Nonlinear Fitting Results of Isotherm Models^a^

Langmuir model	*q*_max_ (mg/g)	*k*_L_ (L/mg)		*R*^2^	RSS	χ^2^
	308.7	0.0154		0.9739	249	27.7

Freundlich model	*k*_F_ ((mg/g) (L/mg)^1/*n*^)	*n*		*R*^2^	RSS	χ^2^
	25.0	2.6		0.9955	42	4.73

R-P model	*k*_R_ (L/g)	*a*_R_ (L^β^/mg^β^)	β	*R*^2^	RSS	χ^2^
	21.9	0.6	0.67	0.9960	38	4.74

a For adsorption, 2 mg of OBSG/2 mL of solution, *c*_0_(U) = 50–900 mg/L, *t* = 1 h, pH = 4.6–4.7, room temperature.

The highest adsorption capacity of OBSG toward U(VI) obtained from the current isotherm experiments is 297.3 mg/g (*c*_0_(U) = 900 mg/L), which is 210% higher than the unmodified BSG (96 mg/g).^30^ In addition, the adsorption capacity of OBSG is higher than those of reported biosorbents and synthetic adsorbents (see Table 3), such as cellulose nanofibers (167 mg/g),^22^ hydrochar (67 mg/g),^8^ and ion-imprinted resin based on carboxymethyl cellulose (U-CMC-SAL, 180 mg/g).^65^ Some adsorbents like the chitosan-derived adsorbent (CTPP)^9^ and graphene oxide-derived adsorbent (CoFe_2_O_4_-rGO)^7^ present a close adsorption capacity (>220 mg/g) to OBSG but need a longer time to reach adsorption equilibrium (72 h for CTPP and 3 h for CoFe_2_O_4_-rGO), while OBSG shows fast adsorption kinetics of only 1 h. In practical application, the decontamination of uranium from wastewater and the natural water environment commonly involves trace U(VI) concentration at the μg/L level instead of the mg/L level.^66^ Therefore, a calculation of the equilibrium concentration (*c*_e_, μg/L) and removal ratio (%) was performed according to the Freundlich isotherm, as determined in Figure 7a, using an initial U(VI) concentration of 50 μg/L within an adsorbent dosage range from 0.25 to 5 g/L. As shown in Figure 7b, for an adsorbent dosage of 2 g/L, the equilibrium concentration of U(VI) is estimated to be lower than 1 μg/L with a removal ratio of 98%. A further increase in the adsorbent dosage (>2 g/L) would result in a removal ratio of over 99%. This indicates that OBSG could be used as a cheap and sustainable alternative to synthetic adsorbents for uranium removal in the real water environment.

**Table 3:** Summary of the Adsorption Capacity of U(VI) onto Different Adsorbents

adsorbent	*q*_max_ (mg/g)	conditions	reference
BSG	96	*m* = 2 mg, *V* = 2 mL, *t* = 2 h, pH = 4.7, *c*_0_ = 100–1000 mg/L, room temperature	(30)
ABSG	220	*m* = 2 mg, *V* = 2 mL, *t* = 1 h, pH = 4.7, *c*_0_ = 100–1000 mg/L, room temperature	(30)
OBSG	297.3	*m* = 2 mg, *V* = 2 mL, *t* = 1 h, pH = 4.6–4.7, *c*_0_ = 50–900 mg/L, room temperature	this study
cellulose nanofibers	167	0.05 wt %, *t* = 2 h, pH = 6.5 ± 0.5, *c*_0_ = 80–1530 ppm	(22)
U-CMC-SAL	180	*m* = 0.03 g, *V* = 30 mL, *t* = 3 h, pH = 5.0, *c*_0_ = 10–400 mg/L, *T* = 30 °C	(65)
CoFe_2_O_4_-rGO	227	*m* = 0.02 g, *V* = 50 mL, *t* = 3 h, pH = 6.0, *T* = 25 °C	(7)
CTPP	237	*m* = 50 mg, *V* = 50 mL, *t* = 72 h, pH = 5.0, *c*_0_ = 100–2000 mg/L, *T* = 25 ± 0.5 °C	(9)
hydrochar	67	*m* = 0.01 g, *V* = 50 mL, *t* = 50 min, pH = 6.0, *c*_0_ = 10–120 mg/L, *T* = 25 °C	(8)

#### 2.3.4 Effect of Temperature on Adsorption

**Figure 8:**
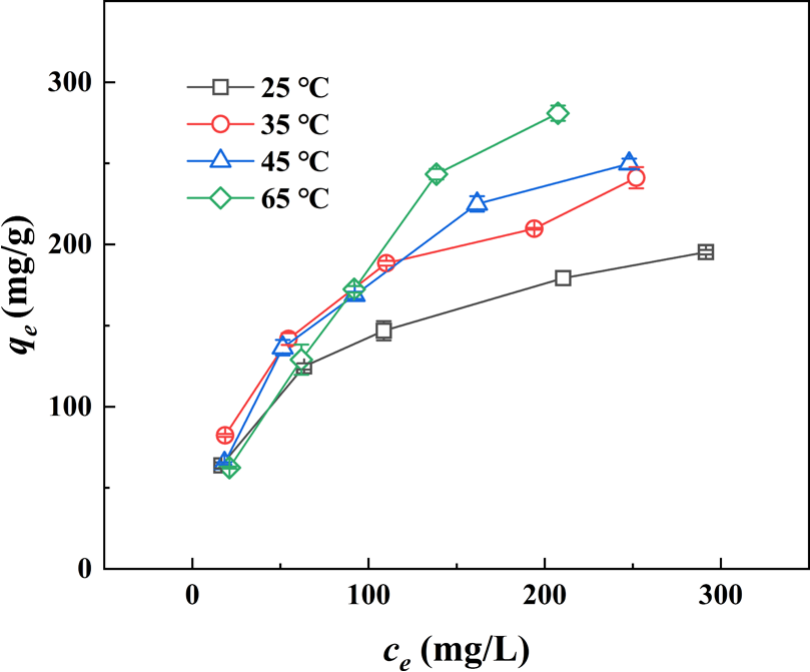
Adsorption isotherms of UO_2_^2+^ onto OBSG at different temperatures. For adsorption, 2 mg of OBSG/2 mL of solution, *c*_0_(U) = 100–500 mg/L, *t* = 1 h, pH = 4.7, temperature = 25–65 °C, stirrer speed = 180 rpm.

The effect of the temperature on UO_2_^2+^ adsorption was investigated by recording adsorption isotherms at 25, 35, 45, and 65 °C (Figure 8). The results show that a rising temperature is conducive for UO_2_^2+^ adsorption onto OBSG. For example, the amount of U(VI) adsorbed on OBSG increases from 195.3 mg/g at 25 °C to 280.9 mg/g at 65 °C at an initial concentration of 500 mg/L. This could result from an increasing number of active sites of swollen OBSG as the temperature rises.^67^ In addition, the adsorption isotherms were also determined in the presence of 0.1 M NaClO_4_ as the supporting electrolyte (Figure S5), showing a decrease in the amount of U(VI) adsorbed on OBSG. This is probably because the supporting electrolyte causes a decrease in the swelling of OBSG, which is also observed for lignocellulose materials (kraft-liner pulps).^68^ Thus, increasing the temperature could no longer promote a rise in the available active sites resulting from the enhanced swelling of OBSG, and the temperature dependency is no longer observed.

### 2.4 Investigation of Adsorption Mechanisms

**Figure 9:**
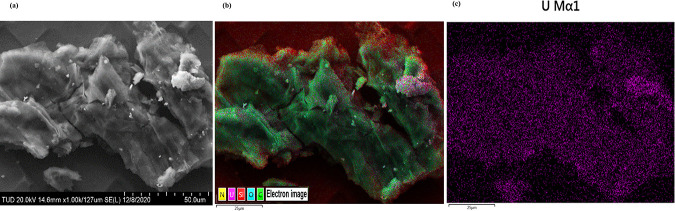
(a) SEM image (magnification of 1000 times) of uranyl ion-loaded OBSG, (b) EDX elemental mapping of uranyl ion-loaded OBSG (20 kV/10 μA, magnification of 1000 times, 25 frames), and (c) distribution of uranium on uranyl ion-loaded OBSG. For ion loading, 50 mg of OBSG/50 mL of solution, pH = 4.7, *c*_0_(U) = 500 mg/L, 1 h, room temperature.

The determined Brunauer–Emmett–Teller (BET) surface area of OBSG is <2 m^2^/g, which is consistent with the literature reporting a BET surface of BSG of 0.48 m^2^/g.^69^ This indicates that oxidation has no influence on the specific surface area of the biosorbent. As OBSG is a non-porous material, the adsorption of UO_2_^2+^ occurs mainly on the surface of OBSG. SEM–EDX analysis provides direct information about the surface morphology of OBSG and the distribution of uranium on OBSG, as shown in Figure 9. Figure 9a confirms that OBSG has a rough surface and an irregular shape with no obvious pore structure. The even distribution of uranium on the OBSG surface (Figure 9b,c) points at numerous carboxyl groups that are equally distributed on the surface of OBSG and strongly interact with the uranyl ions.

**Figure 10:**
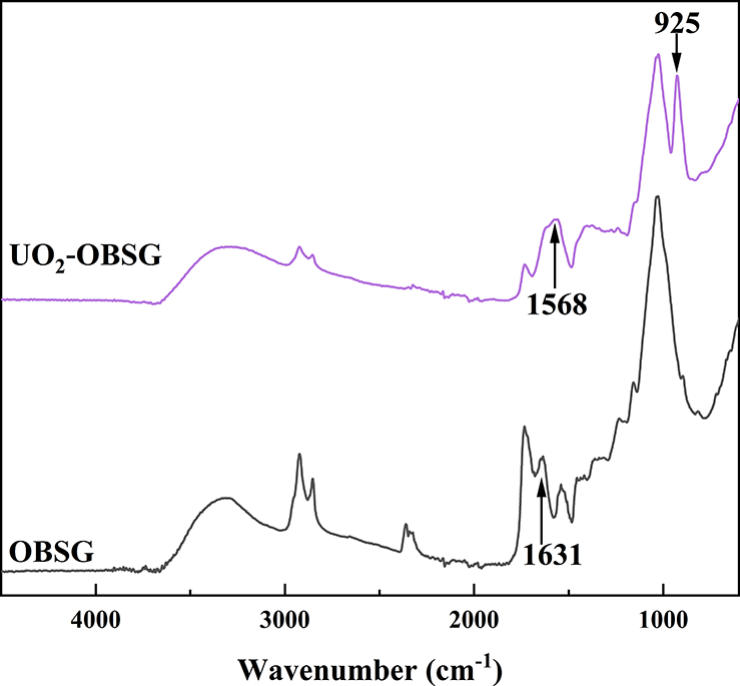
FT-IR spectra of OBSG and UO_2_^2+^-loaded OBSG. For metal loading, 50 mg of OBSG/50 mL of solution, *c*_0_(U) = 500 mg/L, pH = 4.7, 1 h, room temperature.

To get further information about the interactions between functional groups and the adsorbates and to explore the structure of the metal-adsorbent complex, the FT-IR spectra of OBSG before and after UO_2_^2+^ adsorption were recorded (Figure 10). The spectra show a shift of the antisymmetric and symmetric stretching vibrations of −COO^–^ from 1631 and 1451 cm^–1^ (OBSG) to 1568 and 1410 cm^–1^ (UO_2_^2+^-loaded OBSG), which confirms the involvement of carboxyl groups in the adsorption. According to the literature,^70^ one or both oxygen atoms of the carboxyl groups could interact with the metal ions, and the differences between the antisymmetric and symmetric stretching bands (Δυ_as-vs_) could define the complexation model between carboxyl groups and metal ions. Commonly, a value of Δυ_as-vs_ > 200 cm^–1^ indicates a monodentate binding of metal ions by the carboxyl groups, whereas Δυ_as-vs_ < 150 cm^–1^ suggests a bidentate structure. A calculated Δυ_as-vs_ of 158 cm^–1^ upon the adsorption of UO_2_^2+^ suggests a bidentate binding mode. In addition, a new absorption band related to the presence of uranium at 925 cm^–1^ occurs in the spectrum of UO_2_^2+^-loaded OBSG. One possible assignment for this band is the antisymmetric stretching vibration of (UO_2_)_3_(OH)_5_^+^ as this hydrolyzed species occurs in the current pH = 4.7 and is adsorbed onto OBSG.^71^ Another possibility is that this band is attributed to UO_2_(CH_3_COO)_3_^–^, as reported by Müller et al.^72^ In the present study, interactions with several carboxyl groups to UO_2_^2+^or other hydrolyzed species are more likely the cause of the observed absorption band. On the basis of above investigations, the proposed adsorption mechanisms of UO_2_^2+^ onto OBSG are summarized as (a) ion-exchange between UO_2_^2+^ and H^+^ released from carboxyl groups of OBSG through the electrostatic effect and (b) bidentate binding of UO_2_^2+^ with two oxygen atoms of the carboxyl groups.

### 2.5 Desorption and Reusability of Oxidized Brewer’s Spent Grain

**Figure 11:**
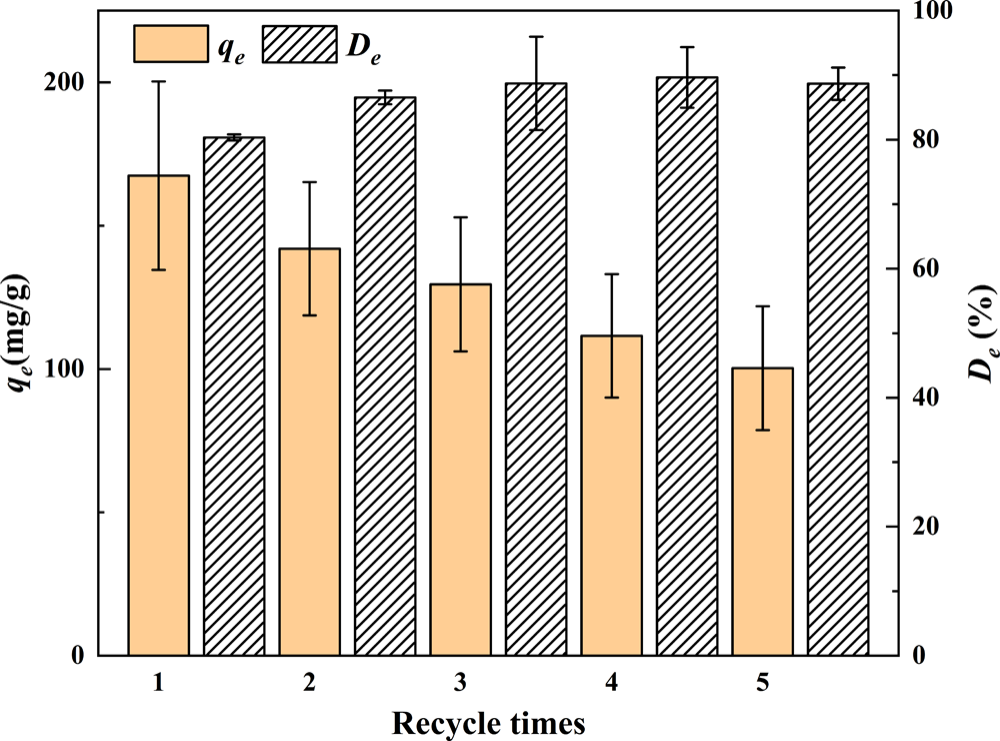
Adsorption capacity and desorption ratio of U(VI) onto OBSG during adsorption–desorption cycles. For adsorption, 1 mg of OBSG/1 mL of solution, *c*_0_(U) = 300 mg/L, pH = 4.7, 1 h, room temperature. For desorption, 5 mg of UO_2_-OBSG/1 mL of 0.5 M HCl, 2 h, room temperature.

The desorption properties and reusability of adsorbents are of great importance for applications as they minimize waste generation and are of economic advantage. In the present study, 0.5 M HCl is applied as a desorption and regeneration agent for UO_2_^2+^-loaded OBSG and the results for five cycles are illustrated in Figure 11. The adsorption capacity of OBSG toward U(VI) decreases gradually during the reuse cycles, from 167.4 to 100.3 mg/g after 5 times of regeneration. This is probably due to the hydrolysis of the biomass under strong acidic conditions and the loss of some surface functional groups.^73^ Meanwhile, the desorption ratio increases after the first cycle and reaches nearly 90% after the third cycle. Although the adsorption capacity is decreased, OBSG still preserves a good adsorption capacity (60% of the original adsorption capacity) with a desorption ratio of 89% after five cycles. Therefore, OBSG could be used for multiple cycles and further reduce the costs and waste production of the adsorption process.

### 2.6 Performance of Oxidized Brewer’s Spent Grain in Simulated Seawater

To explore the practical application of OBSG, the influence of carbonate and high salt content on the adsorption capacity of OBSG has been tested under simulated seawater conditions.^74^ As shown in Table 4, OBSG presents adsorption capacities of 10.8 ± 0.1 mg/g (*c*_U_ = 10 mg/L) and 23.8 ± 0.7 mg/g (*c*_U_ = 30 mg/L) under these conditions. The results are slightly lower than some modified biosorbents reported in the literature at a low concentration of 10 mg/L, for example, functionalized natural cellulose fibers (16 mg/g)^75^ and SSUP fibers (15.1 mg/g).^76^ However, at a higher concentration (30 mg/L), OBSG shows a better performance than some synthetic adsorbents such as pA@Poly(VBC-co-DVB) (14.8 mg/g)^77^ and IIP polymer (15.3 mg/g).^78^ These results show an interesting potential of using OBSG for uranium adsorption in seawater conditions.

**Table 4:** Comparison of the U(VI) Adsorption Capacity in Simulated Seawater Conditions

adsorbent	*q*_e_ (mg/g)	conditions	reference
OBSG	10.8 ± 0.1	*m*/*V* = 0.2 g/L, *c*_U_ = 10 mg/L, 193 mg/L NaHCO_3_, 25.6 g/L NaCl, pH = 7.7, *t* = 16 h	this study
OBSG	23.8 ± 0.7	*m*/*V* = 0.2 g/L, *c*_U_ = 30 mg/L, 193 mg/L NaHCO_3_, 25.6 g/L NaCl, pH = 7.7, *t* = 16 h	this study
functionalized natural cellulose fibers	16	*m*/*V* = 0.2 g/20 mL, *c*_U_ = 10 mg/L, pH = 8.2, r.t., *t* = 48 h	(79)
SSUP fibers	15.1	*c*_U_ = 8 mg/L, simulated seawater	(80)
pA@Poly(VBC-*co*-DVB) (beads)	14.8	*c*_U_ = 50 mg/L, pH = 8, in artificial seawater	(81)
IIP polymer	15.3	*c*_U_ = 200 mg/L, pH 9, with Na_2_CO_3_	(82)

## 3 Conclusions

Although research on cellulose-based biosorbents for uranium adsorption has been carried out over the past decades, it remains challenging to find a renewable and readily applicable raw material. The current study shows for the first time the successful oxidation of BSG with 85 wt % H_3_PO_4_ and NaNO_2_, which leads to an increase in the content of the carboxyl groups from 0.15 to 1.3 mmol/g in OBSG. OBSG demonstrates fast adsorption kinetics in 1 h and an adsorption capacity for U(VI) of 297.3 mg/g (*c*_0_(U) = 900 mg/L, pH = 4.7), which is superior to other biosorbents reported in the literature. Further studies using FT-IR reveal that the possible adsorption mechanisms rely on the ion-exchange effect of UO_2_^2+^ and H^+^ released from the carboxyl groups and the complexation of UO_2_^2+^ with the two oxygen atoms of the carboxyl groups. For practical application, adsorption–desorption studies show that OBSG retains 60% of the original adsorption capacity with an 89% desorption ratio after five adsorption–desorption cycles. The evaluation of OBSG performance in simulated seawater conditions and low concentrated uranyl ion solution indicates a potential usage in low concentration, high salt content, and the presence of carbonate. The results show that BSG could be used as a low-cost and easily available raw material for nitro-oxidation to produce an effective uranium adsorbent without pre-treatment. This may reduce the cost and waste generation when treating uranium-contaminated water. In addition, the present study provides information on the modification of functional groups in protein-rich biomass, which can give inspiration to explore this approach for similar biomass.

## 4 Materials and Methods

### 4.1 Materials

H_3_PO_4_ (85 wt %, Fisher Scientific), NaNO_2_ (>99 wt %, Fisher Scientific), UO_2_(CH_3_COO)_2_·2H_2_O (Merck KGaA), HCl (37 wt %, VWR Chemicals), H_2_O_2_ (35 wt %, Carl Roth GmbH), KMnO_4_ (>99 wt %, Fisher Scientific), NaOH (97 wt %, VWR Chemicals), NaNO_3_ (99.5 wt %, Grüssing GmbH), HF (40 wt %, Merck KGaA), H_3_BO_3_ (99.9 wt %, Alfa Aesar), Na_2_CO_3_ (99.5 wt %, Grüssing GmbH), NaHCO_3_ (99 wt %, Grüssing GmbH), and HNO_3_ (super quality 69 wt %, Carl Roth GmbH) were applied as purchased. Ultrapure water (18.2 MΩ cm, arium pro, Sartorius) was used in all experiments.

### 4.2 Preparation of Standardized Brewer’s Spent Grain

Brewer’s spent grain (BSG, water content 78 wt %) was obtained from our laboratory-scale brewery plant (Technical University of Dresden, Germany) during the production of a Pilsner beer directly after the mashing process. Pilsner malt (14.6 kg, Weyermann) was used in this brewing. During the mashing process, 53 L of water was poured initially, with a replenishment volume of 58 L. The temperature and time of different mashing procedures are summarized in Table S9. The fresh BSG was then stored at −16 °C until further processing. For the preparation of standardized BSG, the material was defrosted at room temperature and dried at 60 °C under reduced pressure (<70 mbar) for 72 h to reduce the water content to less than 5 wt %. Afterward, BSG was milled using a coffee grinder (MayOcean) for 30 s, left to rest for 10 s, and milled again for 20 s. The milled BSG was sieved into three different fractions (>710, 315–710, and <315 μm) for oxidation. For general adsorption studies and characterization, the fraction smaller than 315 μm (designated as BSG) with a water content of 3.0 wt %, a N content of 5.1 wt %, and an estimated protein content of ∼29.5 wt % was used.

### 4.3 Oxidation of Brewer’s Spent Grain

The oxidation of BSG was performed at room temperature by stirring 1 g of standardized BSG with 16 mL of 85 wt % H_3_PO_4_ and 0.8 g of NaNO_2_ (Figure S6) in a 100 mL Erlenmeyer flask using a magnetic stirrer (IKA, RCT basic) at a stirrer speed of 140 rpm for 10 min followed by reacting for another 16 h without stirring. After that, 50 mL of cold ultrapure water was added to quench the reaction. The oxidized BSG was washed with ultrapure water and filtered repeatedly until the pH of the filtrate reached 5. To explore other possible oxidation methods for BSG, H_2_O_2_ and KMnO_4_ were also tested as oxidants according to the literature with modifications. For the H_2_O_2_ method,^34,35^ 2 g of BSG was mixed with 20 mL of ultrapure water and 0.4 mL of 1 M HCl at 100 °C. Afterward, 10 mL of 35 wt % H_2_O_2_ was added into the mixture dropwise and the mixture was refluxed at 100 °C for 2 h. For oxidation using KMnO_4_,^36^ 1 g of BSG was stirred with 0.18 g of KMnO_4_ and 20 mL of 0.15 M H_2_SO_4_ at 60 °C for 2 h. Then, the oxidation products were filtered and washed with ultrapure water repeatedly. All the materials obtained from the three different oxidation methods were dried at 60 °C under reduced pressure (<70 mbar) to reduce the water content to less than 5 wt %. For general adsorption studies and characterization, nitro-oxidized BSG obtained from the fraction smaller than 315 μm (designated as OBSG) with a water content of 3.6 wt %, a N content of 1.1 wt %, and an estimated protein content of 6.4 wt % was employed.

### 4.4 Adsorption Studies

Batch adsorption experiments of UO_2_^2+^ were performed by suspending 2 mg of the adsorbent in 2 mL of the uranyl acetate solution of the required concentration in micro centrifuge tubes (2 cm^3^, Safe-Lock, Eppendorf) using an overhead shaker (Reax 2, Heidolph) with a rotation speed of 60 rpm at room temperature. To determine the optimum pH for adsorption, 1.0 mol/L or 0.1 mol/L HNO_3_ was used to adjust the initial pH to the range of 1–5, which was carefully chosen to prevent precipitation. The equilibrium pH was measured with an InLab micro pH electrode (Mettler Toledo). For the kinetics study, a series of adsorption experiments were performed at different time intervals (0–120 min) at pH = 4.7 with a constant initial U(VI) concentration of 300 mg/L. The adsorption experiments were performed in duplicate, and both the average value and standard deviation are reported. The adsorption isotherm was obtained using different initial concentrations of U(VI) ranging from 50 to 900 mg/L at pH = 4.6–4.7 for 1 h. The isotherm experiments were performed in triplicate, and both the average value and standard deviation are reported. After adsorption, the solution was filtered using a 13 mm syringe filter with a 0.22 μm PTFE film (Fisher Scientific), and the mass concentration (mg/L), which refers to the elemental U content before and after adsorption, was determined using inductively coupled plasma–optical emission spectrometry (ICP-OES) (OPTIMA 2000DV, PerkinElmer, USA). The adsorption capacity (*q*_e_, mg/g) was calculated using the following equation (eq 7):
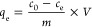
7where *c*_0_ (mg/L) and *c*_e_ (mg/L) are the metal concentrations before and after adsorption, *m* (g) is the mass of the adsorbent, and *V* (L) is the volume of metal solution.

The effects of temperature on the adsorption capacity of OBSG were examined by performing adsorption isotherms at different temperatures, namely, 25, 35, 45, and 65 °C. Generally, 2 mg of OBSG was mixed with 2 mL of uranyl ion solution with different initial concentrations of U(VI) (100–500 mg/L) at pH = 4.7 in a 10 mL test tube using a magnetic stirrer (IKA, RCT basic) at a stirrer speed of 180 rpm. The temperature was controlled by a circulation thermostat (UH 4, MLW-Medingen). The experiments were performed in duplicate, and both the average value and standard deviation are reported.

The desorption and reusability of OBSG were examined through five adsorption–desorption cycles. Therefore, 50 mg of OBSG was added into 50 mL of 300 mg/L U(VI) solution at pH = 4.7 and shaken with an overhead shaker for 1 h at room temperature. After adsorption, the mixture was centrifuged, and the supernatant was analyzed for the remaining U(VI) concentration. The UO_2_^2+^-loaded OBSG was washed once with ultrapure water and dried at 60 °C under reduced pressure (<70 mbar) for 12 h. Afterward, UO_2_-OBSG was weighed again, suspended with 0.5 M HCl as desorption agent with an adsorbent/acid ratio of 5 mg/mL for 2 h. The regenerated OBSG was centrifuged, and the supernatant was collected for ICP-OES analysis. The OBSG was washed three times (ultrapure water) and then dried at 60 °C for 12 h before the next cycle. The desorption ratio *D*_e_ (%) was calculated as follows (eq 8):
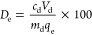
8where *c*_d_ (mg/L) is the U(VI) concentration after desorption, *V*_d_ (L) is the volume of HCl, *m*_d_ (g) is the mass of UO_2_-loaded OBSG for desorption, and *q*_e_ (mg/g) is the adsorption capacity of OBSG determined every cycle. All adsorption and desorption experiments were performed in triplicate, and both the average value and standard deviation are reported.

Adsorption experiments of OBSG under simulated seawater conditions were performed according to the literature with modifications.^74^ Simulated seawater consists of 25.6 g/L NaCl, 193 mg/L NaHCO_3_, and 10 or 30 mg/L U(VI). For adsorption, 2 mg of OBSG was mixed with 10 mL of simulated seawater at pH = 7.0 (30 mg/L U(VI)) or 7.7 (10 mg/L U(VI)) in 15 mL centrifuge tubes using an overhead shaker with a rotation speed of 60 rpm for 16 h at room temperature. The experiments were carried out in duplicate, and both the average value and standard deviation are reported.

### 4.5 Characterization and Analysis Methods

Infrared (FT-IR) spectra were obtained with a single-beam Fourier transform infrared VERTEX 70 spectrometer (Bruker). An ATR (attenuated total reflectance) unit (diamond) with single-reflection optics at an interaction angle of 45° was used. The spectra were recorded over the range of 4500–600 cm^–1^ with a resolution of 4 cm^–1^ and averaged over 32 scans. To investigate the detailed changes of chemical structures, the spectra were processed using the OPUS software package as provided by Bruker to compare the intensity of certain bands. Baseline corrections were applied at 3000, 2875, 1769, 1573, 1191, and 857 cm^–1^. Then, the spectra were normalized with respect to −CH_2_– antisymmetric stretching vibration bands (∼2924 cm^–1^). For normalization, the absorbance value of this band was set to 1.0 and the complete spectrum was multiplied accordingly. The raw spectra are provided in Figure S7. For the adsorption mechanism study, raw FT-IR spectra of OBSG and UO_2_-loaded OBSG were used.

^13^C solid-state NMR spectra were recorded on a BRUKER Ascend 800 MHz spectrometer using a commercial 3.2 mm MAS NMR probe and operating at a resonance frequency of 201.2 MHz. The MAS frequency was 15 kHz. Adamantane was used as an external standard. Ramped ^1^H-^13^C cross-polarization (CP, contact time: 4 ms) and SPINAL ^1^H-decoupling during the signal acquisition were applied. The recycle delay was 3 s. 26,000 scans were accumulated for BSG and OBSG.

Scanning electron microscopy (SEM) and energy-dispersive X-ray spectroscopy (EDX) analyses were performed on a scanning electron microscope (SU8020, HITACHI) equipped with an energy-dispersive X-ray spectrometer X-Max^N^ (OXFORD Instrument) at an electron beam voltage of 20 kV. The U(VI)-loaded OBSG sample was dried at 60 °C under reduced pressure (<70 mbar) for 48 h before the measurement. The surface morphology images were taken at a magnification of 1000 times, and the EDX elemental mapping was taken at 20 kV/10 μA and a magnification of 1000 times for 25 frames.

The thermogravimetric (TG) analysis of the biosorbents was performed by a simultaneous thermal analyzer (STA 8000, PerkinElmer). The samples were heated from 40 to 600 °C with a heating rate of 20 °C/min under a helium atmosphere. The contents of Ca, Fe, Mn, Mg, Zn, K, Na, P, and Si (mineral elements) of BSG and OBSG were determined by ICP-OES after microwave-assisted digestion. Generally, 3 mL of HNO_3_ (supra pure, 69 wt %) and 2 mL of HCl (37 wt %) were added into ca. 0.05 g of biosorbents. After 1 h at room temperature, 1 mL of HF (40 wt %) was added, and the mixture was vortexed. After standing for another 1 h, 10 mL of saturated H_3_BO_3_ was added into the mixture to complex the HF before heating for 10 min at 170 °C (MARS 6, CEM GmbH). Elemental analysis was performed on a Vario MICRO cube (Elementar Analysatorsysteme GmbH) in CHNS mode to determine the contents of carbon, nitrogen, hydrogen, and sulfur. The oxygen content was calculated by mass balance considering the contents of carbon, nitrogen, hydrogen, sulfur, and the mineral elements determined by ICP-OES. The results of elemental analysis and mineral element analysis are provided in Tables S2 and S3. The protein content was estimated according to the N content by multiplying by a factor of 5.83.^79^ The chemical composition of BSG is given in Table S10.

The point of zero charge (pH_pzc_) of BSG and OBSG was determined by the solid addition method^80^ using 0.2 g of the adsorbent suspended in 10 mL of 0.1 M NaNO_3_ solution. The initial pH value (pH_0_) of the solution was adjusted to 1–10 using 0.1 M HNO_3_ or 0.1 M NaOH. The equilibrium pH (pH_e_) was recorded after mixing for 16 h, and the change of pH (ΔpH) was calculated. The pH_pzc_ was determined by plotting ΔpH versus pH_0_, and the pH_pzc_ is equal to the pH_0_ value when ΔpH = 0.

The content of oxygen functional groups (OFGs) was quantified using Boehm titration.^81^ In general, a mixture of 0.9 g of adsorbents and 50.00 mL of one of the three reaction bases, NaHCO_3_, Na_2_CO_3_, and NaOH (0.05 M) was shaken for 24 h. The mixtures were filtered, and three 10.00 mL aliquots were taken for titration. The NaHCO_3_ and NaOH samples were acidified with 20.00 mL of 0.05 M HCl, whereas for Na_2_CO_3_ samples, 30.00 mL of 0.05 M HCl was added. The acidified solutions were then put into an ultrasonic bath (Sonorex RK 52H, Bandelin electronic GmbH & Co. KG) for 20 min to expel dissolved CO_2_ and titrated with 0.05 M NaOH using a phenolphthalein indicator. The amount of amine groups (−NH_2_) was determined using a volumetric method according to the literature.^82^ The adsorbent (0.1 g) was suspended in 50 mL of 0.05 M HCl for 16 h, and the remaining amount of HCl was titrated with 0.05 M NaOH using a phenolphthalein indicator.

## Supporting Information Available

Elemental analysis and mineral element contents of BSG and OBSG, pH value of uranyl ion solution before and after adsorption, effect of the rotation speed on the adsorption kinetics, kinetic model fitting results at a rotation speed of 80 rpm, statistical results of comparing pseudo-first-order and pseudo-second-order kinetic models (at 60 and 80 rpm), statistical results of comparing the R-P model and Freundlich model, adsorption isotherms at different temperatures with 0.1 M NaClO_4_, parameters of the mashing process to produce BSG, chemical composition of BSG, distribution diagram of uranyl acetate solution depending on pH, adsorption capacity of La^3+^ in the dependence of the amount of NaNO_2_ used in the oxidation of BSG, raw and normalized FT-IR spectra of oxidized products by different oxidation methods, raw FT-IR spectra of BSG and OBSG (<315 μm), and FT-IR spectra of BSG and OBSG with and without D^+^ labeling (PDF)
