# Supplementary material for: Recycling of Brewer’s Spent Grain as a Biosorbent by Nitro-Oxidation for Uranyl Ion Removal from Wastewater
Source: ACS Omega. 2021 Jul 19;6(30):19364–77. doi: 10.1021/acsomega.1c00589 (PMC8340112; doi:10.1021/acsomega.1c00589)
Supplement: Supplementary file 1 — ao1c00589_si_001.pdf [file ao1c00589_si_001.pdf]

Supporting information

Recycling of brewer's spent grain as biosorbent by nitro-oxidation for uranyl ions removal from wastewater

Yi Su[a], Marco Wenzel[a], Silvia Paasch[b], Markus Seifert[a], Wendelin Böhm[c], Thomas Doert[d], Jan J. Weigand[a]*

[a] Chair of Inorganic Molecular Chemistry, TU Dresden, 01062 Dresden, Germany

[b] Chair of Bioanalytical Chemistry, TU Dresden, 01062 Dresden, Germany

[c] Chair of Food Chemistry, TU Dresden, 01062 Dresden, Germany

[d] Chair of Inorganic Chemistry II, TU Dresden, 01062 Dresden, Germany

---

*Corresponding author

E-mail address: jan.weigand@tu-dresden.de (J.J. Weigand)

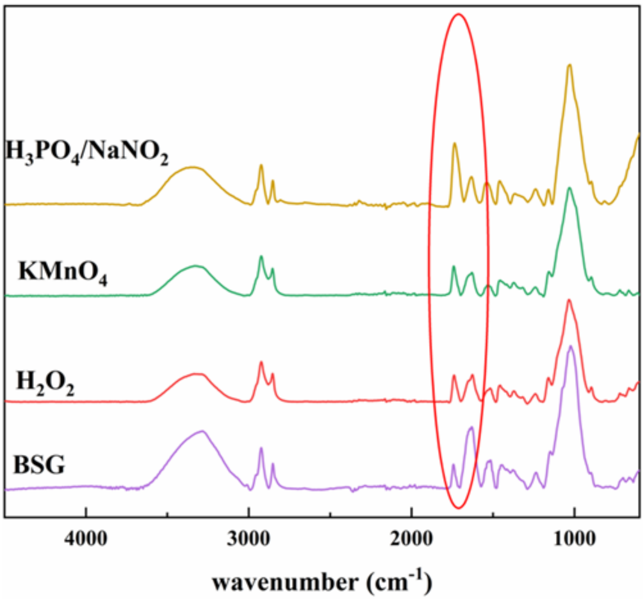

Figure S1. FT-IR spectra of oxidized products by different oxidation methods. H_2O_2 method: 2 g BSG, 10 mL 35 wt % H_2O_2, 0.4 mL 1 M HCl, reflux at 100 °C for 2 h. $KMnO_4$ method: 1 g BSG, 0.18 g $KMnO_4$, 20 mL 0.15 M H_2SO_4 at 60 °C for 2 h. $H_3PO_4/NaNO_2$ method: 1 g BSG, 16 mL 85 wt % H_3PO_4, 0.8 g $NaNO_2$ at room temperature for 16 h.

The ν(COOH) vibration at 1742 cm^{-1} for BSG and 1732 cm^{-1} for OBSG has been confirmed by the shifts upon D^+ labelling. Typically, 30 mg adsorbents were mixed with 2 mL D_2O in 2 mL centrifuge tube for 64 h. Then the mixture was centrifuged, the supernatant was discarded, and the obtained solid was dried at 60 °C for 12 h. The D^+ labelling samples are named as BSG-D_2O and OBSG-D_2O and subjected to FT-IR measurement using a single beam Fourier transform infrared VERTEX 70 spectrometer

(Bruker). The entire spectra were recorded over the range of 4500 to 600 cm^{-1} with a resolution of 4 cm^{-1} and averaged over 32 scans. Within the particularly interested area (2000–1200 cm^{-1}), detailed spectra with a resolution of 1 cm^{-1} and averaged over 64 scans were also measured (Figure S2).

Table S1. Shifts of ν(COOH) vibration in BSG and OBSG FT-IR spectra upon D^+ labelling.

	ν(origin)	ν(D_2O)	Δν
BSG	1742 cm^{-1}	1739 cm^{-1}	3 cm^{-1}
OBSG	1732 cm^{-1}	1729 cm^{-1}	3 cm^{-1}

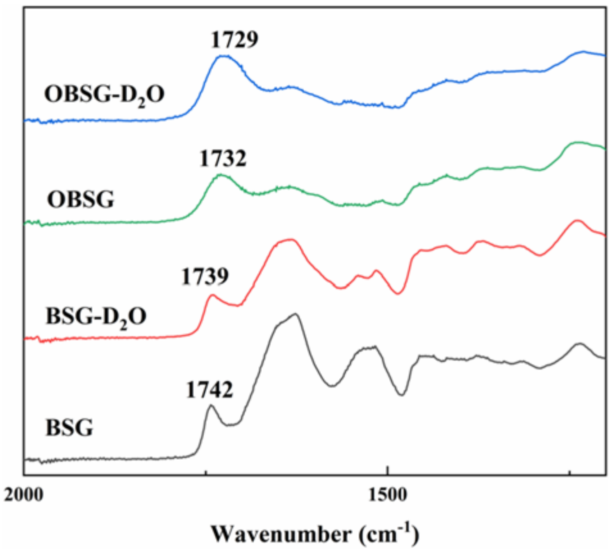

Figure S2. FT-IR spectra of BSG and OBSG with or without D^+ labelling. For D^+ labelling: 30 mg adsorbents/ 2 mL D_2O for 64 h; for IR measurement: resolution = 1 cm^{-1}, averaged over 64 scans.

Table S2. Elemental analysis of BSG and OBSG.

	N (%)	C (%)	H (%)	S (%)	Mineral (%)	O (%)
BSG	5.1 ± 0.1	49.1 ± 0.1	6.1 ± 0.2	0.3 ± 0.1	1.4 ± 0.1	38.0 ± 0.6
OBSG	1.1 ± 0.1	42.6 ± 0.8	5.4 ± 0.1	0.1 ± 0.1	1.0 ± 0.1	49.8 ± 1.2

Table S3. Mineral elements content (mg/kg) of BSG and OBSG.

	K	Na	P	Ca	Fe	Mg	Mn	Zn	Si	Mineral (%)
BSG	808 ± 0.2	9 ± 1	5284 ± 3	1096 ± 2	0	1487 ± 0.2	0	0	5184 ± 0.4	1.4 ± 0.1
OBSG	0	0	760 ± 30	1.1 ± 0.1	0	0	0	0	9400 ± 270	1.0 ± 0.1

Errors are those obtained from the ICP measurements.

Table S4. Comparison of pH value of uranyl ions solution before and after adsorption.

Experiment Number	pH (before)	pH (after)
1	4.7	4.2
2	3.9	3.2
3	2.8	2.7
4	2.3	2.3
5	1.2	1.3

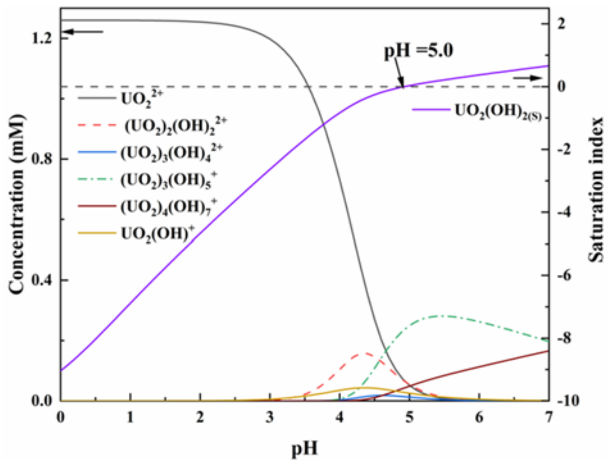

Figure S3. Distribution diagram of uranyl acetate solution. For input data, $c_0(U)$ = 300 mg/L = 1.26 mM, $c(CH_3COO^-)$ = 2.52 mM, alkalinity: no specification, pH = 0–7.0, increment = 0.1, calculated by Visual MINTEQ 3.1.[1]

Table S5. Results of statistical methods using for comparing pseudo-first-order and pseudo-second-order kinetic model (rotation speed = 60 rpm).

Preferred model	Model Name				
AIC	Pseudo-first-order kinetic model				
BIC	Pseudo-first-order kinetic model				
F-Test	Pseudo-first-order kinetic model				
Akaike Information Criterion (AIC)[1]	*RSS*	*N*	*p*	*AIC*[2]	*Akaike's weights*[3]
Pseudo-first-order kinetic model	3.0747	8	2	4.3501	0.9999
Pseudo-second-order kinetic model	29.9194	8	2	22.5525	0.0001
Bayesian Information Criteria (BIC) test[4]	*RSS*	*N*	*p*	*BIC*[5]	ΔBIC
Pseudo-first-order kinetic model	3.0747	8	2	−1.4116	0
Pseudo-second-order kinetic model	29.9194	8	2	16.7909	18.2025
F-Test[6]	*RSS*	*DF*		*F*[7]	Prob > F
Pseudo-first-order kinetic model	3.0747	6		0.1028	0.9930
Pseudo-second-order kinetic model	29.9194	6			

1. Pseudo-first-order kinetic model has lower *AIC* value and so is more likely to be correct. This model is 8966.14 times more likely to be correct.

2. $AIC = Nln\left(\frac{RSS}{N}\right) + 2p + \frac{2p(p+1)}{N-p-1}$, where *N* is the number of data points and p is the number of free parameters to be estimated.

3. $Akaike's\ weight = \frac{e^{-0.5\Delta AIC}}{1+e^{-0.5\Delta AIC}}$, where ΔAIC is the deference between two *AIC* values.

4. Pseudo-first-order kinetic model has lower *BIC* value and so is more likely to be correct. BIC difference (ΔBIC) more than 10 gives decisive conclusion that pseudo-first-order kinetic model is correct.

5. $BIC = Nln\left(\frac{RSS}{N}\right) + pln(N)$, and ΔBIC is the deference between two *BIC* values.

6. At the 0.05 significance level, pseudo-first-order kinetic model is more likely to be correct.

7. $F = RSS_1/RSS_2$, where RSS_1 and RSS_2 are the residual sum of squares of pseudo-first-order kinetic and pseudo-second-order kinetic model, respectively.

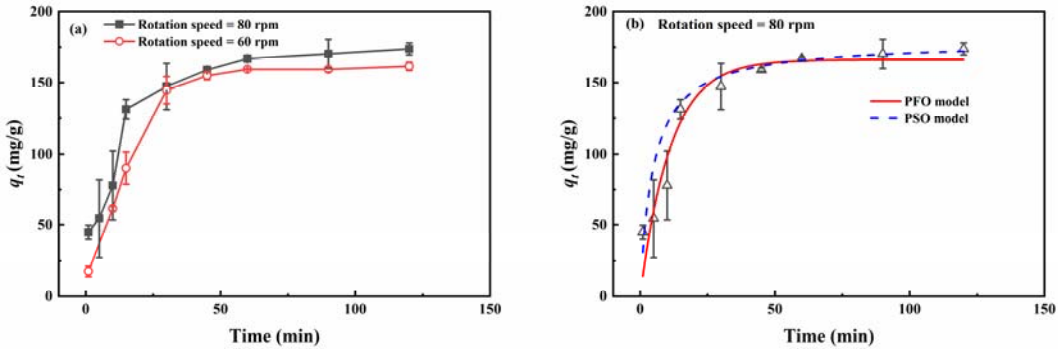

Figure S4. (a) Effect of rotation speed on the adsorption kinetics. For adsorption, 2 mg OBSG/ 2 mL solution, c_0(U) = 300 mg/L, pH = 4.7, room temperature, and (b) non-linear fitting of pseudo-first-order (PFO) and pseudo-second-order (PSO) kinetic model of the kinetic data for rotation speed = 80 rpm.

Table S6. Parameters of kinetic models at higher rotation speed. For adsorption, 2 mg OBSG/ 2 mL solution, c_0(U) = 300 mg/L, pH = 4.7, rotation speed = 80 rpm, room temperature.

Pseudo-first-order kinetic model	k_1 (min^{-1})	$q_{e,exp}$ (mg/g)	$q_{e,cal1}$ (mg/g)	R^2	RSS	χ^2
	0.0888	167.0	166.2	0.9107	60.0	8.6
Pseudo-second-order kinetic model	k_2 (g·mg^{-1}·min^{-1})	$q_{e,exp}$ (mg/g)	$q_{e,cal2}$ (mg/g)	R^2	RSS	χ^2
	0.0012	167.0	179.0	0.9692	20.7	3.0

Table S7. Results of statistical methods using for comparing pseudo-first-order and pseudo-second-order kinetic model (rotation speed = 80 rpm).

Preferred model	Model Name				
AIC	Pseudo-second-order kinetic model				
BIC	Pseudo-second-order kinetic model				
F-Test	Pseudo-second-order kinetic model				
Akaike Information Criterion (AIC)[1]	*RSS*	*N*	*p*	*AIC*[2]	*Akaike's weight*[3]
Pseudo-first-order kinetic model	60.0214	9	2	27.8759	0.0082
Pseudo-second-order kinetic model	20.6690	9	2	18.2827	0.9918
Bayesian Information Criteria (BIC) test[4]	*RSS*	*N*	*p*	*BIC*[5]	ΔBIC
Pseudo-first-order kinetic model	60.0214	9	2	23.6676	9.5932
Pseudo-second-order kinetic model	20.6690	9	2	14.0744	0
F-Test[6]	*RSS*	*DF*		*F*[7]	Prob > F
Pseudo-first-order kinetic model	60.0214	7		2.9039	0.0915
Pseudo-second-order kinetic model	20.6690	7			

1. Pseudo-second-order kinetic model has lower *AIC* value and is more likely to be correct. This model is 121.101 times more likely to be correct.
2. $AIC = Nln\left(\frac{RSS}{N}\right) + 2p + \frac{2p(p+1)}{N-p-1}$, where *N* is the number of data points and *p* is the number of free parameters to be estimated.
3. $Akaike's\ weight = \frac{e^{-0.5\Delta AIC}}{1+e^{-0.5\Delta AIC}}$, where ΔAIC is the deference between two *AIC* values.
4. Pseudo-second-order kinetic model has lower *BIC* value and is more likely to be correct. ΔBIC gives a strong support to pseudo-second-order kinetic model.
5. $BIC = Nln\left(\frac{RSS}{N}\right) + pln(N)$, and ΔBIC is the deference between two *BIC* values.
6. At the 0.05 significance level, pseudo-second-order kinetic model is more likely to be correct.
7. $F = RSS_1/RSS_2$, where RSS_1 and RSS_2 are the residual sum of squares of pseudo-first-order kinetic and pseudo-second-order kinetic model, respectively.

Table S8. Results of statistical methods using for comparing R-P model and Freundlich model.

Preferred model	Model Name				
AIC	Freundlich model				
BIC	Inconclusive				
F-Test	Freundlich model				
Akaike Information Criterion (AIC)[1]	*RSS*	*N*	*p*	*AIC*[2]	*Akaike's weight*[3]
Freundlich model	42.5480	11	2	24.3087	0.8790
R-P model	37.9033	11	3	28.2752	0.1210
Bayesian Information Criteria (BIC) test[4]	*RSS*	*N*	*p*	*BIC*[5]	ΔBIC
Freundlich model	42.5480	11	2	22.0738	0
R-P model	37.9033	11	3	23.2001	1.1264
F-Test[6]	*F*[7]	df_1-df_2	df_2	Prob > F	
	0.9803	1	8	0.3511	

1. Freundlich model has lower *AIC* value and is more likely to be correct. This model is 7.2665 times more likely to be correct.
2. $AIC = Nln\left(\frac{RSS}{N}\right) + 2p + \frac{2p(p+1)}{N-p-1}$, where *N* is the number of data points and *p* is the number of free parameters to be estimated.
3. $Akaike's\ weight = \frac{e^{-0.5\Delta AIC}}{1+e^{-0.5\Delta AIC}}$, where ΔAIC is the deference between two *AIC* values.
4. Freundlich model with lower *BIC* seems to be correct. BIC difference (ΔBIC) less than 2 is inconclusive.
5. $BIC = Nln\left(\frac{RSS}{N}\right) + pln(N)$, and ΔBIC is the deference between two *BIC* values.
6. At the 0.05 significance level, Freundlich model (simple model) is more likely to be correct.
7. $F = \frac{(RSS_1 - RSS_2)/(df_1 - df_2)}{RSS_2/df_2}$, where RSS_1 and df_1 are the residual sum of squares and degrees of freedom of the simple model (Freundlich model), respectively, and RSS_2 and df_2 are the residual sum of squares and degrees of freedom of the complex model (R-P model), respectively.

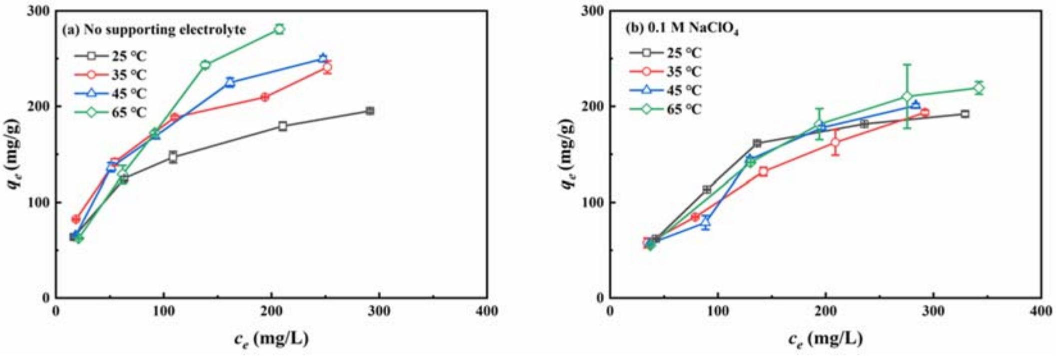

Figure S5. Adsorption isotherms of U(VI) onto OBSG at different temperature (a) without supporting electrolyte and (b) with 0.1 M $NaClO_4$. For adsorption: 2 mg OBSG/ 2 mL solution, $c_0(U)$ = 100−500 mg/L, t = 1 h, pH = 4.7, temperature = 25–65 °C, stirrer speed = 180 rpm.

Table S9. Parameters of the mashing process to produce BSG.

Procedure	Initial temperature (°C)	End temperature (°C)	Time (min)
Mash-in	55	54.8	10
Protein rest	62	61.8	30
Maltose rest	68	67.8	10
Sugar rest	72	71.8	25
Mash-out	78	77.8	10

Table S10. Chemical composition of BSG.

Water (%)	Ash (%)	Acid insoluble lignin (%)	Acid soluble lignin (%)	Lignin (%)	Protein (%)	Cellulose (%)	Other (%)
3.0	3.6	32.5 ± 3.8	0.34 ± 0.01	32.8 ± 3.8	29 ± 0.6	8.75 ± 0.8	22.8 ± 5.2

Methods: The water content and ash content (20 °C/min, Air, 40–850 °C) of BSG was obtained from TG analysis. The protein content was estimated according to the N content by multiplying by a factor of 5.83.

The cellulose content was measured using the method proposed by Updegraff[3,4] according to literature with minor modification. Generally, four replicates of 20 to 40 mg BSG were weighed in 6 mL glass tubes with screw caps. After adding 3 mL of a mixture of acetic acid/water/nitric acid (8/2/1, v/v/v), the suspension was heated in a boiling water bath for 30 min with occasional mixing. After cooling in an ice bath, the tubes were centrifuged for 10 min at 1000 *g* and the supernatant was discarded. The pellet was thoroughly re-suspended in 5 mL bidestilled water and centrifuged again.

The washing process was repeated once more, and the supernatant was discarded. The remaining pellet was then incubated with 2.5 mL of 72 wt % sulphuric acid for 1 h with vortex every 5–10 min. The clear solution was transferred into a 10 mL volumetric flask and filled to the marking with water (after sufficient cooling time). For the photometric determination of cellulose, 20 µL aliquots were diluted to 400 µL with water and 1 mL of ice-cold 100 mg anthrone in 50 mL sulphuric acid (95 wt %) was added. The mixture was heated for 15 min in a boiling water bath, cooled in an ice bath for 2 min and let stand at room temperature for 10 min prior to photometric measurement at 620 nm against reagent blank. A cellulose stock solution for calibration was prepared by dissolving 57.4 mg of microcrystalline cellulose in 10 mL of 72 wt % sulphuric acid and diluting to 500 mL with water. Dilutions in the range of 2.87 µg to 28.7 µg in 400 µL water were prepared in duplicate and processed by anthrone assay as mentioned above.

The content of lignin was determined according to ASTM E1758−01 and method reported by Balogun et.al[5]. More specifically, 300 ± 10 mg BSG was incubated in 3 mL 72 wt % H_2SO_4 for 1 h at 30 °C, then diluted into 4 wt % H_2SO_4, and subjected to a secondary hydrolysis in an autoclave reactor (DAB-3, Berghof Products+Instruments GmbH) at 121 °C for 1 h. The hydrolyzed mixture was filtrated, and the obtained solid was weighted. The content of acid-insoluble lignin was obtained by subtracting the ash content from the solid content. Meanwhile, the filtrate was collected for the measurement using a UV-vis spectrometer (Lambda 25, PerkinElmer) with a quartz

cuvette (10 mm) in the range of 200–250 nm with a resolution of 0.1 nm. The acid-soluble lignin content was calculated according to the absorbance at 205 nm using an absorption coefficient of 110 L g^{-1} cm^{-1}.

Preliminary experiment employing La^{3+} adsorptions were used to optimize the dosage of NO_2^- per g BSG in the oxidation. Typically, 1 g standardized BSG was oxidized using 16 mL of 85 wt % H_3PO_4 and different amounts of $NaNO_2$ (0, 0.2 g, 0.4 g, 0.6 g, 0.8 g, 1.0 g) at room temperature for 16 h. As shown in the following Figure S3, the adsorption capacity of La^{3+} onto the oxidized BSG increases as the dosage of $NaNO_2$ increases from 0 to 0.8 g/g BSG, and further increase of the amount of $NaNO_2$ added to 1.0 g/g BSG results in no obvious improvement of the adsorption capacity. Thus, a 0.8 g $NaNO_2$ per g BSG were used in the oxidation of BSG.

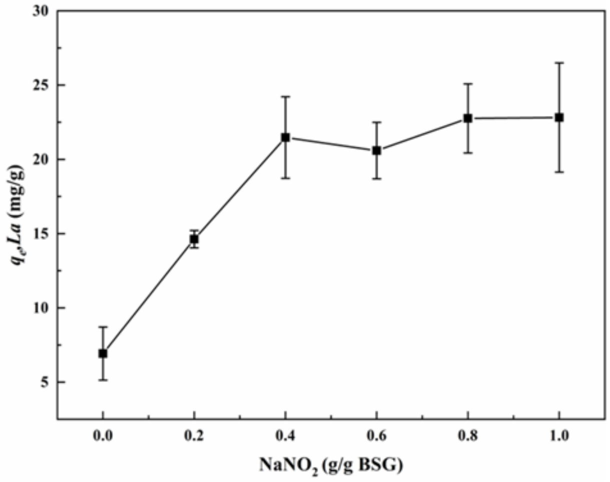

Figure S6. Adsorption capacity of La^{3+} in dependence of the amount of $NaNO_2$ used in the oxidation of BSG. For adsorption: 2 mg adsorbent/ 2 mL solution, $c_0(La^{3+})$ = 100 mg/L, pH = 5.5, 2 h, room temperature.

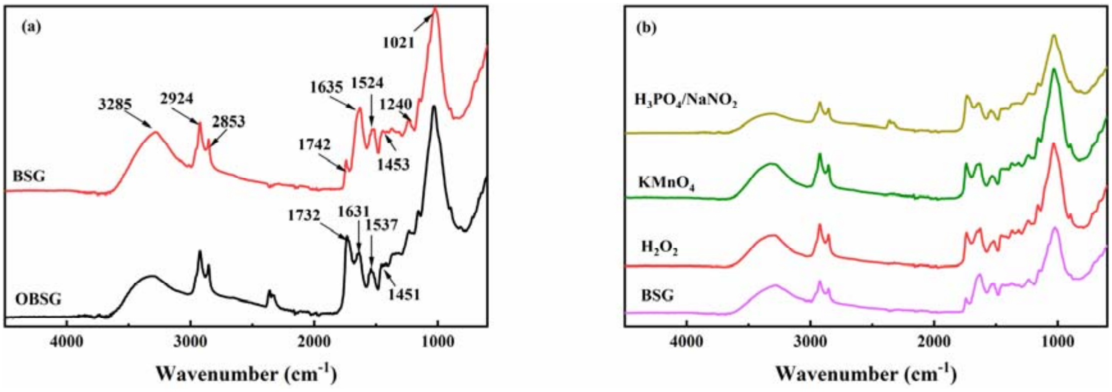

Figure S7. (a) Raw FT-IR spectra of BSG and OBSG (<315 μm) and (b) Raw FT-IR spectra of oxidized products by different oxidation methods. H_2O_2 method: 2 g BSG, 10 mL 35 wt % H_2O_2, 0.4 mL 1 M HCl, reflux at 100 °C for 2 h. $KMnO_4$ method: 1 g BSG, 0.18 g $KMnO_4$, 20 mL 0.15 M H_2SO_4 at 60 °C for 2 h. $H_3PO_4/NaNO_2$ method: 1 g BSG, 16 mL 85 wt % H_3PO_4, 0.8 g $NaNO_2$ at room temperature for 16 h.

Reference:

1 J. P. Gustafsson, Visual MINTEQ ver. 3.1, Department of Land and Water Resources Engineering, KTH, SE-100 44, Stockholm, Sweden; available at the URL https://vminteq.lwr.kth.se/download/.

2 M. G. Adamson, Chemical thermodynamics of uranium, *J. Nucl. Mater.*, 1993, **200**, 154–155.

3 D. M. Updegraff, Semimicro determination of cellulose inbiological materials, *Anal. Biochem.*, 1969, **32**, 420–424.

4 S. Bauer and A. B. Ibáñez, Rapid determination of cellulose, *Biotechnol. Bioeng.*, 2014, **111**, 2355–2357.

5 A. O. Balogun, F. Sotoudehniakarani and A. G. McDonald, Thermo-kinetic, spectroscopic study of brewer's spent grains and characterisation of their pyrolysis products, *J. Anal. Appl. Pyrolysis*, 2017, **127**, 8–16.
